# Unsupervised learning of temporal features for word categorization in a spiking neural network model of the auditory brain

**DOI:** 10.1371/journal.pone.0180174

**Published:** 2017-08-10

**Authors:** Irina Higgins, Simon Stringer, Jan Schnupp

**Affiliations:** 1 Department of Experimental Psychology, University of Oxford, Oxford, England; 2 Department of Physiology, Anatomy and Genetics (DPAG), University of Oxford, Oxford, England; Georgia State University, UNITED STATES

## Abstract

The nature of the code used in the auditory cortex to represent complex auditory stimuli, such as naturally spoken words, remains a matter of debate. Here we argue that such representations are encoded by stable spatio-temporal patterns of firing within cell assemblies known as polychronous groups, or PGs. We develop a physiologically grounded, unsupervised spiking neural network model of the auditory brain with local, biologically realistic, spike-time dependent plasticity (STDP) learning, and show that the plastic cortical layers of the network develop PGs which convey substantially more information about the speaker independent identity of two naturally spoken word stimuli than does rate encoding that ignores the precise spike timings. We furthermore demonstrate that such informative PGs can only develop if the input spatio-temporal spike patterns to the plastic cortical areas of the model are relatively stable.

## Introduction

The nature of the neural code used by the auditory brain to represent complex auditory stimuli, such as naturally spoken words, remains uncertain [1, 2]. A variety of spike rate and spike timing coding schemes are being debated. Rate encoding presumes that the identity of an auditory stimulus is encoded by the average firing rate of a subset of neurons, but the precise timing of individual spikes is irrelevant. Temporal encoding suggests that different auditory stimuli are represented by spatio-temporal patterns of spiking activity within populations of neurons, where the relative timing of the spikes is part of the representation.

A widely held view of the auditory pathway is that temporal encoding plays a major role in the early subcortical areas, but becomes increasingly less important in the midbrain and the cortical areas [3]. Here we build on existing theories of learning in spiking neural networks [4–7] to argue that temporal coding may have a crucial role to play in the auditory cortex. In particular, we argue that the basic information encoding units for representing complex auditory stimuli, such as naturally spoken words, in the auditory cortex are spatio-temporal patterns of firing within cell assemblies called polychronous groups (PGs) [6].

**Fig 1:**
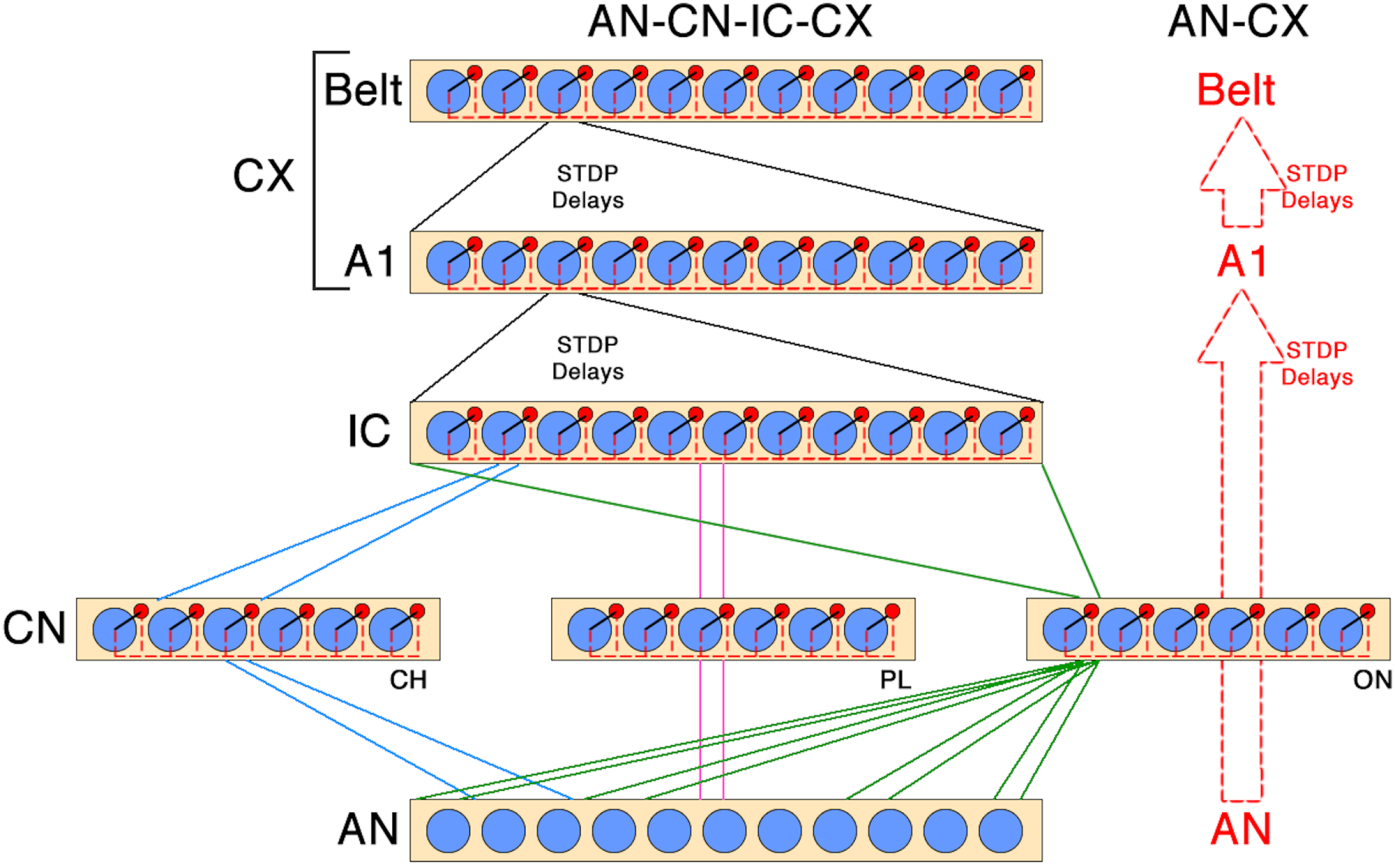
Schematic representation of the full AN-CN-IC-CX and reduced AN-CX models. In the AN-CX model, direct plastic connections from the AN to the A1 replace CN and IC layers. Blue circles are excitatory, red circles inhibitory cells. Solid black lines within a layer represent one-to-one connections from each excitatory neuron to a corresponding inhibitory neuron. Dashed red lines within a layer correspond to one-to-all connections from each inhibitory neuron to all excitatory neurons. For between-layer connections, the different colours have the following meaning: blue corresponds to the tonotopic connectivity of chopper neurons, red corresponds to the one-to-one connectivity of the primary-like neurons, and green corresponds to the sparse connectivity over a wide frequency range of the onset neurons. There is full IC-A1 and A1-Belt connectivity with STDP and a distribution of conduction delays.

Our hypothesis is evaluated using a biologically inspired hierarchical spiking neural network model of the auditory brain comprising of the auditory nerve (AN), cochlear nucleus (CN), inferior colliculus (IC), and auditory cortex (CX) stages, where the last CX stage includes primary (A1) and “higher order” (Belt) cortex (Fig 1). Using only biologically plausible local spike-time dependent plasticity (STDP) learning [8], our auditory brain model is trained to discriminate between two naturally spoken words, “one” and “two”, in a speaker independent manner through unsupervised exposure to the stimuli. In order to succeed on the task, the model has to learn how to cope with the great variability of the acoustic waveforms of these sounds when pronounced by many different speakers (TIDIGITS database [9]).

We show that stable spatio-temporal patterns of firing (PGs) spontaneously emerge within the CX stage of the model, and that they are significantly more informative of the auditory object identity than an alternative rate coded information encoding scheme that disregards the precise spike timing information. Furthermore, our results show that such PG-based learning in the plastic cortical stages of the model relies on relatively stable spatio-temporal input patterns. Due to the stochasticity of spiking times in the AN, if the AN spike patterns are fed directly to the plastic cortical areas, these spike patterns are not stable enough for the emergence of informative PGs in the cortical areas [10]. Hence, the particular subcortical circuitry of the CN and IC is necessary to reduce the high levels of noise known to exist in the AN and re-introduce stability within the firing patterns that serve as input to the plastic cortical areas of the model, thus enabling PG-based learning to emerge in the plastic CX [10]. In summary, the main contributions of our paper are the following:

1. We develop a hierarchical, physiologically motivated spiking neural network model of the auditory brain that is able to learn to differentiate between two naturally spoken words in a speaker-independent manner
2. Due to its biological plausibility, our model can be used to make neurophysiologically testable predictions, and thus lead to further insights into the nature of the neural processing of auditory stimuli
3. Here we use our biologically plausible neural network to provide simulation evidence to argue for spatio-temporal information encoding using polychronous groups (PGs) [6] in the auditory cortex
4. We demonstrate that PG-based learning in the plastic auditory cortex relies on the relative stability of the input spatio-temporal firing patterns

## Materials and methods

### Learning mechanisms

In order to form speaker independent representations of different words, the auditory brain has to be able to respond in a manner that discriminates between different words but not between different exemplars of the same word. This is a challenging task, given the great variability in the raw auditory waves corresponding to the same word due to differences in pronunciation both within and between speakers. This input variability is further compounded by the stochasticity present in the firing patterns generated at the first neural stage of auditory processing, the AN. How can the brain discover the statistical regularities differentiating various words in such noisy inputs? We believe an answer to this question can be found in a number of independent yet overlapping theories describing how spiking neural networks with local STDP learning may discover and amplify the statistical regularities in temporal input patterns [4–7].

**Fig 2:**
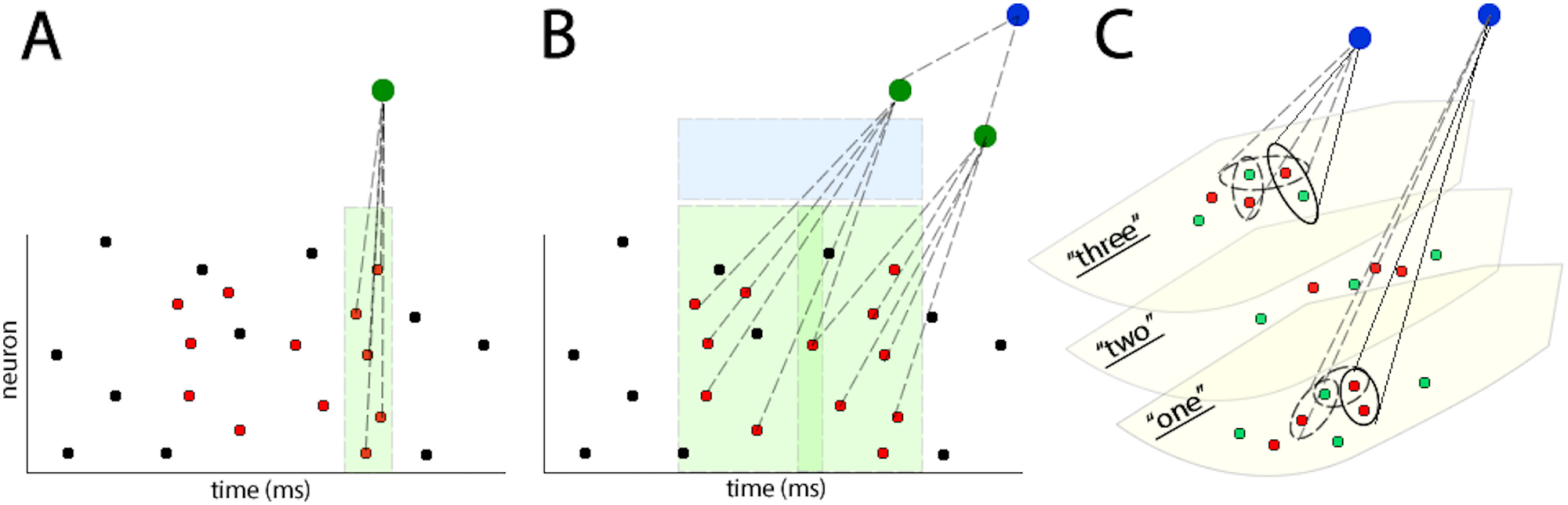
**A**: single output neuron (green) can learn to pick out a repeating spatio-temporal pattern of firing (red) out of statistically identical noise (black) [5]. In this example the output neuron relies on concurrent input from at least five input neurons in order to fire. Due to instantaneous axonal conduction, the neuron has a very narrow temporal integration window of a few milliseconds (shown in light green). **B**: If random axonal conduction delays (Δ_*ij*_ in our models, see Table 1) are added for the feedforward connections ($w_{ij}^{BL}$ in our models, see Table 1), each neuron in the next stage of the model (green) becomes sensitive to a particular pattern of firing in the input. Axonal conduction delays extend temporal integration windows of output neurons (shown in light green). Adding extra output layers with random distributions of axonal delays creates a hierarchy of pattern learning neurons. Neurons at the end of such a hierarchy (blue) have the largest temporal integration windows (shown in light blue) **C**: different pronunciations of words “one”, “two”, and “three” by two different speakers (red and green dots respectively) lie on different low dimensional manifolds. Polychronous groups (PGs) [6] in the auditory cortex (blue circles) can learn to become sensitive to similar pronunciations of one preferred word (solid ovals). Continuous transformation learning [4] extends the sensitivity of PGs to more different pronunciations of the preferred word (dashed ovals), while maintaining the selectivity of PGs to exemplars of one word only.

**Learning to extract repeating patterns from noise**: The first relevant idea was described by [5], who showed that a single spiking neuron can learn to pick out a repeating spatio-temporal pattern of firing from statistically identical noise using STDP learning (see Fig 2A). This effect depends on the particular biologically realistic STDP learning configuration with stronger long term depression (LTD) (parameters *α*_*d*_ and *τ*_*d*_ in our models, see Table 1) compared to long term potentiation (LTP) (parameters *α*_*p*_ and *τ*_*p*_ in our models, see Table 1). Such STDP configuration results in the overall weakening of the feedforward connections ($w_{ij}^{BL}$ in our models, see Table 1) to the output neuron due to the random input firing in response to noise. This is true for all connections apart from those originating from the input cells involved in the repeating pattern, which instead get strengthened due to repeating LTP every time the pattern is presented. Such simple circuits are able to cope with variable or degraded inputs while maintaining stable, informative output representations. For example, they may learn to cope with the presence of temporal jitter in the input on the order of a few milliseconds, additive Poisson noise of up to 10 Hz, or loss of up to half of the neurons participating in the input repeating pattern [5].

**Table 1:** Parameters used for the full AN-CN-IC-CX and reduced AN-CX models of the auditory brain (where these differ for the LTD magnitude (*α*_*d*_), the AN-CX parameters are given as second values following a slash). AN—auditory nerve; CN—cochlear nucleus with three subpopulations of cells discriminated based on their connectivity: chopper (CH), primary-like (PL) and onset (ON); IC—inferior colliculus; A1—primary auditory cortex; Belt—belt area of the auditory cortex. The parameters were found to be optimal using a grid search heuristic on a two vowel recognition task (see [10] for details). This parameter search resulted in the best model performance when no inhibitory recurrent connections were present in the CH and PL subpopulations of the CN ($w_{ij}^{EI}=w_{ij}^{IE}=0$ nA). The sparse connectivity parameter for the AN to ON connections defines the proportion of dead synapses between these two layers.

AN-CN-IC-CX/AN-CX Model Parameters								
	*Within Layer Parameters*							
	**Layer 1**	**Layer 2a**	**Layer 2b**	**Layer 2c**	**Layer 3**	**Layer 4**	**Layer 5**	
**Corresponding Brain Area**	AN	CH (CN)	PL (CN)	ON (CN)	IC	A1 (CX)	Belt (CX)	
**Excitatory Cell Number**	1000	1000	1000	100	1000	1000	1000	
**Inhibitory Cell Number**	0	1000	1000	100	1000	1000	1000	
**E→I Connection Strength**, *$w_{ij}^{EI}$* (nA)	N/A	0	0	1000	1000	1000	1000	
**I→E Connection Strength**, *$w_{ij}^{IE}$* (nA)	N/A	0	0	−75	0	−6	−6	
	*Between Layer Parameters*							
	**AN-CH**	**AN-PL**	**AN-ON**	**CH-IC**	**PL-IC**	**ON-IC**	**IC/AN-A1**	**A1-Belt**
**Axonal Delays**, Δ*_*ij*_* (ms)	N/A	N/A	N/A	N/A	N/A	N/A	[0, 50]	[0, 50]
**Connectivity** (neurons)	Gaussian (*σ* = 26)	1-to-1	sparse (0.46)	Gaussian (*σ* = 2)	1-to-1	full	full	full
**Initial Connection Strength**, *$w_{ij}^{BL}$* (nA)	[25, 30]	1000	26	400	400	3	[30, 35]	[30, 35]
**Spike Rule**	N/A	N/A	N/A	N/A	N/A	N/A	nearest	nearest
**STDP Rule**	N/A	N/A	N/A	N/A	N/A	N/A	mixed	mixed
**LTP Time Constant**, *τ*_*p*_ (ms)	N/A	N/A	N/A	N/A	N/A	N/A	15	15
**LTD Time Constant**, *τ*_*d*_ (ms)	N/A	N/A	N/A	N/A	N/A	N/A	25	25
**LTP Magnitude**, *α*_*p*_	N/A	N/A	N/A	N/A	N/A	N/A	0.005	0.005
**LTD Magnitude**, *α*_*d*_	N/A	N/A	N/A	N/A	N/A	N/A	−0.015/−0.033	−0.015/−0.033
**Max Synaptic Strength**, *w*_*max*_ (nA)	N/A	N/A	N/A	N/A	N/A	N/A	60	60

**Extending the memory capacity and temporal receptive fields for repeating pattern learning**: The output neuron described by [5] can learn one short spatio-temporal pattern of firing (see Fig 2A). The learning depends on firing co-occurences within the pattern that lie within the neuron’s temporal integration window (on the order of a few milliseconds). Such a setup in its original form therefore has limited value for learning longer spatio-temporal patterns, such as words, that can last on the order of hundreds of milliseconds. This shortcoming can be partially averted by the addition of randomly initialised conduction delays (Δ_*ij*_ in our models, see Table 1) for the feed-forward connections ($w_{ij}^{BL}$ in our models, see Table 1). The conduction delays would extend the temporal range of coincidences that each output neuron can detect (see Fig 2B).

If an output neuron receives input spikes through connections with randomly initialised delays, it will only fire if the right input neurons fire in the right temporal order that matches the delay lines. Only then would their spikes arrive at the output neuron coincidentally and depolarise it enough to fire. Since different output neurons will have different axonal delays initialised for their afferent connections, they will be sensitive to different input patterns of firing. Hence, the addition of extra output neurons with different randomly initialised delays would introduce heterogeneity in the types of spatio-temporal patterns the output layer as a whole can learn (see Fig 2B). Such heterogeneity would allow the feedforward network to organise its firing into a hierarchy of “polychronous groups” (PGs) [6]. PGs are stable spatio-temporal patterns of firing, where neurons within a layer “exhibit reproducible time-locked but not synchronous firing patterns with millisecond precision” [6]. Each neuron can be part of numerous PGs, thus increasing network memory capacity [6]. The idea is that PGs in each layer will be sensitive to particular parts of repeating spatio-temporal patterns that are characteristic of a particular stimulus class. Throughout the hierarchy of the network, PGs will emerge that are more invariant to the different variations of their preferred pattern and have longer temporal receptive fields. The details of such PG-based learning is discussed next.

The nature of learnt PGs is shaped by the interplay between delay lines, STDP learning and stimulus structure. A random distribution of conduction delays sets up a repertoire of PGs in a network as described above. When a stimulus is presented to the network, the resulting input spatio-temporal firing patterns are propagated through a set of connections with random delays. If that set of connections is large then it may contain subsets of connections with delays which happen to match the characteristic spatio-temporal firing patterns in the input in a manner that allows the input spikes to converge synchronously on a receiving neuron. This receiving neuron thereby receives super-threshold activation, and its connections to the input spatio-temporal pattern are strengthened by STDP learning.

In this manner, different output layer neurons become sensitized to the characteristic activity of different patterns of firing in the input layer. The temporal structure of the input stimuli may cause the output layer neurons themselves to generate reproducible spatio-temporal firing patterns, giving rise to “higher order” PGs, which may in turn be learned by the next layer in the network. Such a feedforward hierarchy could take advantage of cumulative delays over several layers of connectivity, enabling PGs to discover regularities in the temporal structure of input stimuli over an ever wider temporal scale (see Fig 2B).

**Extending robustness to pattern variability**: While building on the setup described by [5], PG-based learning is still not quite sufficient to enable a feedforward spiking neural network to form speaker independent representations of naturally spoken words, because it is unable to cope with the high degree of pronunciation variability. To tackle this we introduce the last relevant concept: the Continuous Transformation (CT) learning principle [4]. CT learning corresponds to Hebbian learning with an additional constraint on the nature of the data, whereby in expectation over the whole dataset it is imporant that the nearest neighbours of each exemplar of a particular object class in the raw sensory input space are other exemplars of the same object class. This condition is met when the observed data is generated using factors of variation that are densely sampled from their respective continuous distributions. CT learning is a mechanism originally developed to describe geometric transform invariant visual object recognition in a rate-coded neural network model of the ventral visual stream [11]. It takes advantage of the fact that when visual objects undergo smooth transformations, such as rotations, translations or scalings, the nearest neighbours of the resulting projections into the two-dimensional retinal input space have a high degree of overlap or correlation. CT learning binds these similar input patterns together into an invariant representation of that object (or an object orbit according to [12]) and maps them onto the same subset of higher stage neurons.

CT learning can be intuitively thought of as simply a label given to Hebbian learning applied to inputs with highly overlapping neighbouring within-class exemplars. A model with Hebbian learning trained on stimuli with high similarity between the neighbouring transforms of each stimulus class (thus operating using the CT principle) would learn a different type of class boundary compared to the same model trained on stimuli that do not satisfy the CT learning constraints. In the latter case, the model will learn to act as a k-means clustering algorithm. A model operating under the CT learning constraints, however, can learn class boundaries of arbitrary shapes (a more detailed description of CT learning for vision can be found in [13]).

While originally conceived around learning transform invariant representations of visual objects, CT learning generalizes to other modalities where the nearest neighbours of different exemplars of a given stimulus class strongly overlap in the high dimensional raw sensory input space. For example, different pronunciations of the same word might form a low-dimensional manifold for that word, and different words might form different manifolds (see Fig 2C). Two different pronunciations of one word may have highly overlapping AN spatio-temporal firing rasters, and hence be nearest neighbours on the corresponding low-dimensional manifold for that word. By chance, a PG may be sensitive to a particular region of that word manifold. CT learning would then “expand” the span of such a PG to a more extensive region of the manifold, while preserving the selectivity of the PG to its preferred word manifold only. In this way a hierarchy of PGs would emerge through learning in a feed-forward spiking neural network, whereby PGs higher up in the hierarchy would learn to respond to an increasing number of pronunciations of the same word, while ignoring the pronunciations of all other words.

### Stimuli

Recordings of two pronunciations of the digits “one” and “two” from each of 94 native American English speakers served as stimuli (TIDIGITS database [9]). Each utterance was normalised for loudness using the root mean square measure of the average power of the signal. This was done to remove any potential effect of stimulus loudness on learning. The model was trained on the first utterance by each speaker, and tested on the second. The training set was presented to the model ten times. The two digits were presented in an interleaved fashion. Informal tests demonstrated that on average the order in which the stimuli were presented did not significantly affect the performance of the trained models. It did, however, introduce higher trial to trial variability. Hence, we fixed the presentation schedule for the simulations described in this paper for a more fair model comparison. Each word was followed by 100 ms of silence. The silence was introduced to aleviate the confounding problem of having to perform word segmentation.

All reported results are calculated using model responses to the witheld test set of 188 distinct auditory stimuli (2 words spoken by the same 94 speakers as during training, but the particular pronunciations of the words were different from the training set). Each testing exemplar was presented 4 times, because due to the stochasticity of AN responses, input AN spike patterns in response to repeated presentations of the same word were not identical. This means that the results are reported in response to 752 testing examples in total (2 words * 94 speakers * 4 presentations).

### Spiking neural network architecture

To investigate whether information about auditory objects, such as words, is better encoded within spatio-temporal PGs rather than through rate encoding, we constructed a biologically grounded, unsupervised spiking neural network model of the auditory pathway, as shown in Fig 1 (for full model parameters see Table 1) [10]. The AN-CN-IC-CX model comprised of five layers: 1) the auditory nerve (AN); 2) the cochlear nucleus (CN), encompassing subpopulations representing three major ventral CN cell classes described by neurophysiologists: chopper (CH), onset (ON) and primary-like (PL); 3) the midbrain (inferior colliculus or IC) on which all CN subpopulations converge; and 4-5) cortical (CX) layers: primary (A1) and secondary (Belt) auditory cortex. Excitatory CN neurons have been categorised based on their morphology and temporal discharge characteristics in response to short supra-threshold tones into primary-like (PL) (47%), chopper (CH) (36%), onset (ON) (10%) and other (7%) [14, 15]. These major distinct classes of excitatory neurons within the CN are modelled within the corresponding PL, CH and ON subpopulations of the CN in our model. The particular connectivity and response properties of the subcortical layers (CN, IC) play a key role in reducing the physiological noise inherent to the AN which enables our model to learn about the two stimulus classes in a speaker independent manner. [10] demonstrated how an equivalent multi-stage hierarchical model *without* this specific artchitecture of the CN and IC stages was unable to learn to discriminate between simple vowel stimuli unlike the AN-CN-IC-CX model. To verify the importance of the CN and IC layers we also constructed and evaluated a reduced AN-CX model without the CN or IC stages on the digit recognition task presented in this paper.

In the brain sub-populations of the CN do not necessarily synapse on the IC directly. Instead, they pass through a number of nuclei within the superior olivary complex (SOC). The nature of processing done within the SOC in terms of auditory object recognition (rather than sound localisation), however, is unclear. The information from the different CN sub-populations does converge in the IC eventually, and for the purposes of the current argument we model this convergence as direct. The same simplified connectivity pattern (direct CN-IC projections) was implemented by [16] for their model of the subcortical auditory brain.

Another simplification within our models has to do with the higher stages IC, A1 and Belt. They are not as neurophysiologically detailed as AN or CN. For example, IC is a loose approximation of the SOC, IC and MGN; while the A1 and Belt stages of the full AN-CN-IC-CX and the reduced AN-CX models are supposed to be a loose and simplified approximation of the MGN and higher auditory cortical areas in the real brain. We implement full rather than tonotopic connectivity for these layers because tonotopy becomes less pronounced throughout the hierarchy of the higher auditory cortical areas, and we chose to simplify this into the full connectivity for the IC-CX stages of our models. In theory informative PGs could develop with tonotopic connectivity, however in this case they may require more feedforward hierarchical stages before learning to span the full auditory frequency range. Alternatively the addition of recurrent connections within the cortical stages of our model could result in informative PGs that span the whole auditory frequency range without requiring a deep hierarchy of stages. We did not include recurrent connectivity in the cortical stages of our models however. This simplification was done to be able to analyse the emergence and the nature of PGs for auditory stimulus encoding. Detecting PGs is a non-trivial problem even in feedforward architectures. It becomes even harder once recurrency is introduced. While we believe that recurrent connections are important for learning longer auditory sequences, and for dealing with speech in noise, we leave their inclusion for future work.

### Auditory nerve (AN)

The AN comprised of 1000 medium spontaneous rate fibres modelled as described by [17], with log spaced characteristic frequencies (CF) between 300-3500 Hz, and a 60 dB threshold. The AN model by [17] has a high level of physiologically realism, ensuring that our models receive input which is highly representative of the signal processing challenges faced by the real auditory brain hierarchy. AN fibers, both biological ones and those of the model, are noisy channels plagued by “temporal and spatial jitter”. Temporal jitter arises when the propensity of the AN fibers to phase lock to temporal features of the stimulus is degraded by poisson-like noise in the nerve fibers and refractoriness [18]. “Spatial jitter” refers to the fact that neighbouring AN fibers have almost identical tuning properties, so that an action potential that might be expected at a particular fiber at a particular time may instead be observed in neighbouring fibers (the “volley principle” [19]). Both forms of jitter disrupt the firing pattern precision required for PG learning, and reducing the jitter should help the plastic CX layers of the model to learn the statistical structure of the stimulus set [10]. Jitter reduction can be accomplished by the CN and IC layers, which were modelled closely on anatomical and physiological characteristics of their biological counterparts [10].

### Neuron model

Apart from the AN, which was modelled as described by [17], all other cells used in this paper were spiking neurons as specified by [20, 21]. The spiking neuron model by [21] was chosen because it is effectively as computationally efficient as integrate-and-fire neurons, but it comes with a selection of carefully tuned parameters to provide a collection of various verified biologically realistic response properties. This variability of biologically plausible response properties was important, because it allowed us to easily implement different functional responses of neurons within the different stages of the auditory brain pathway as discussed next. In theory our models may perform in a qualitatively similar fashion if implemented using leaky integrate-and-fire units with carefully tuned parameters (such as time constants, synaptic currents and time scales) for different neuron types.

We implemented our models using the Brian simulator with a 0.1 ms simulation time step [22]. The exponential STDP learning rule with *nearest mixed* (*spike rule* and *STDP rule* parameters respectively in Table 1) weight update paradigm was used [22]. See Section “STDP implementation” for the detailed description of the STDP implementation. A range of conduction delays (Δ_*ij*_) between layers is a key feature of our models. In real brains, these delays might be axonal, dendritic, synaptic or due to indirect connections [23], but in the model, for simplicity, all delays were implemented as axonal. The Δ_*ij*_ ∈ [0, 50] ms range was chosen to approximately match the range reported by [6].

The spiking behaviour of Izhikevich’s neurons is governed by the following equations:

$$\begin{array}{c} \frac{dv_{i}}{dt}=0.04{\left(v_{i}\left(t\right)\right)}^{2}+5v_{i}\left(t\right)+140-u_{i}\left(t\right)+I_{i}\left(t\right) \end{array}$$ (1)

$$\begin{array}{c} \frac{du_{i}}{dt}=a\left(b\cdot v_{i}\left(t\right)-u_{i}\left(t\right)\right) \end{array}$$ (2)

where *v*_*i*_(*t*) is the membrane potential of the post-synaptic neuron *i* measured on a mV scale, *u*_*i*_(*t*) is the membrane recovery variable of the post-synaptic neuron *i*, which accounts for the activation of K^+^ and closure of Na^+^ ionic currents, and *t* is time measured on a ms scale. *I*_*i*_(*t*) represents a combination of incoming externally injected ($I_{i}^{ext}\left(t\right)=0$ nA for the simulations described in this thesis) and synaptic currents for the post-synaptic neuron *i* according to the following formula:

$$\begin{array}{c} I_{i}\left(t\right)=\sum_{j}\sum_{l}w_{ij}\delta \left(t-\Delta t_{ij}-t_{j}^{l}\right)+I_{i}^{ext}\left(t\right) \end{array}$$

where *w*_*ij*_ is the magnitude of the synaptic connection strength between the pre-synaptic neuron *j* and the post-synaptic neuron *i*, and $\delta (t-\Delta t_{ij}-t_{j}^{l})$ is the Dirac delta function evaluated at time *t* minus the magnitude of the axonal conduction delay Δ_*ij*_ between the pre- and with the post-synaptic cells *j* and *i* and minus the times of the pre-synaptic spike train $t_{j}^{l}$ indexed by *l*. Dirac delta function is defined as the following:

$$\begin{array}{c} \delta \left(x\right)=\{\begin{array}{c} \infty \;\;\;\;\;\;\;\;\;\;\;\;\;if\;\;\;x=0\;\;\\ 0\;\;\;\;\;\;\;\;\;\;\;\;\;\;\;otherwise \end{array}\;\;\;\;\;\;\;\;where,\int_{-\infty }^{+\infty }\delta \left(x\right)dx=1 \end{array}$$

The after-spike resetting is governed by the following equations:

$$\begin{array}{c} if\;v_{j}\left(t\right)\geq 30\;mV,\;\;then\;\{\begin{array}{c} v_{j}\left(t\right)\leftarrow c\;\;\;\;\;\;\;\;\;\;\;\;\;\\ u_{j}\left(t\right)\leftarrow u_{j}\left(t\right)+d \end{array} \end{array}$$ (3)

where *c* accounts for the after-spike reset of cell membrane potential caused by the fast high-threshold K^+^ conductances, and *d* accounts for the after-spike reset of the membrane recovery variable caused by the slow high-threshold K^+^ and Na^+^ conductances. Parameters *a* − *d* are dimensionless and were adjusted as described next to achieve the required spiking behaviour of neurons. The particular parameter values were chosen in a manner so that the model can replicate the functional properties of various neuron types as suggested by the neurophysiological literature. In practice, other values could have resulted in similar functional performance. Hence the particular values chosen were somewhat arbitrary.

#### Excitatory cells

Neurophysiological evidence suggests that many neurons in the subcortical auditory brain have high spiking thresholds and short temporal integration windows, thus acting more like coincidence detectors than rate integrators [3, 24]. We implemented this behaviour using Izhikevich’s Class 1 neurons [21]. All subcortical (CN, IC) excitatory cells were, therefore, implemented as Class 1, but with a higher threshold compared to Izhikevich’s original specification (see Table 2 for details). To take into account the tendency of neurons in the auditory cortex to show strong adaptation under continuous stimulation [25] Izhikevich’s Spike Frequency Adaptation neurons were chosen to model the excitatory cells in the auditory cortex (A1 and Belt) (Table 2).

**Table 2:** Values of the *a* − *d* constants used in Eqs 1–3 for the excitatory and inhibitory neurons used in the model of the auditory brain. *Neuron Type* refers to model neurons reported by [21]. **a**: time scale of *u*(*t*). Smaller values mean slower recovery. **b**: sensitivity of *u*(*t*) to subthreshold fluctuations in *v*(*t*). Larger values result in subthreshold oscillations and low-threshold spiking. **c**: after-spike reset value of *v*(*t*). **d**: after-spike reset value of *u*(*t*).

Parameter	Excitatory Cortical (Spike Adaptation—F)	Excitatory Subcortical (Class 1—G)	Inhibitory (Phasic bursting—D)
**a**	0.01	0.02	0.02
**b**	0.2	-0.1	0.25
**c**	-65	-55	-55
**d**	8	6	0.05
**Threshold (mV)**	30	30	30

#### Inhibitory cells

Since inhibitory interneurons are known to be common in most areas of the auditory brain [3, 25] except the AN, each stage of the models apart from the AN contained both excitatory and inhibitory neurons. Inhibitory cells were implemented as Izhikevich’s Phasic Bursting neurons [20] (Table 2). The choice of neuron type for inhibitory neurons was motivated by their similarity to resonator [26] rather than integrator [27] neurons due to the Hopf bifurcation characteristic of their dynamics. Preliminary simulations, however, showed that other neuron types can work similarly to the Phasic Bursting type. Sparse connectivity between excitatory to inhibitory cells ($w_{ij}^{EI}$) within a model area was modelled using strong one-to-one connections from each excitatory cell to an inhibitory partner. Each inhibitory cell, in turn, was fully connected to all excitatory cells ($w_{ij}^{IE}$). Such an inhibitory architecture implemented dynamic and tightly balanced inhibition as described in [28], which resulted in competition between excitatory neurons, and also provided negative feedback to regulate the total level of firing within an area. Informal tests demonstrated that the exact implementation or choice of neuron type for within-layer inhibition did not have a significant impact on the results presented in this paper, as long as the implementation still achieved an appropriate level of within-layer competition and activity modulation.

### STDP implementation

The following equations describe the implementation of STDP-based learning within the proposed neural network model of the auditory brain. The weight update is scaled by:

$$\begin{array}{c} f\left(s_{ij}\right)=\{\begin{array}{c} \alpha_{p}e^{-s_{ij}/\tau_{p}},\;\;\;\;\;\;\;if\;s_{ij}>0\;\;\;\;\;\;\;\;LTP\\ \alpha_{d}e^{s_{ij}/\tau_{d}},\;\;\;\;\;\;\;\;\;if\;s_{ij}<0\;\;\;\;\;\;\;\;LTD \end{array} \end{array}$$ (4)

where *τ*_*p*_ and *τ*_*d*_ are STDP time constants, *α*_*p*_ and *α*_*d*_ are constant coefficients, and *s*_*ij*_ is the time difference between a post- and a pre-synaptic spike calculated according to the following:

$$\begin{array}{c} s_{ij}=t_{i}-\left(t_{j}+\Delta_{ij}\right) \end{array}$$

where *t*_*i*_ is the time of the post-synaptic spike, *t*_*j*_ is the time of the pre-synaptic spike, and Δ_*ij*_ is the magnitude of the axonal conduction delay between the pre- and post-synaptic cells. All delays were treated as axonal, whereby each pre-synaptic spike time was set to be the time of spike arrival to the post-synaptic cell, rather than the time of pre-synaptic spike discharge.

Neurophysiological evidence suggests that STDP time constants are asymmetric and are equal to 17 ± 9 ms for LTP and 34 ± 13 ms for LTD [8]. Therefore, the default values of *τ*_*p*_ and *τ*_*d*_ were set to 15 ms and 25 ms correspondingly as suggested by [29].

The equations above calculate a scaling variable *f*(*s*_*ij*_), which were then used to update the synaptic weights. Neurophysiological data suggests that the LTD magnitude is independent of the instantaneous synaptic strength (*w*_*ij*_), while the magnitude of LTP changes inversely proportionally to connection strength, with stronger synapses resulting in less LTP than weaker synapses [8, 30, 31]. These findings are modelled using a mixed STDP paradigm as shown in the equations below, whereby additive learning is used for LTD and multiplicative learning is used for LTP. The minimum bound of 0 nA was set to ensure that the additive LTD did not run away to −∞. The differential equations shown below lead to the bounding of weights in the interval $\left[0,w_{ij}^{max}\right]$.

$$w_{ij}\left(t+1\right)=\{\begin{array}{lll} w_{ij}\left(t\right)+\left(w_{ij}^{max}-w_{ij}\left(t\right)\right)f\left(s_{ij}\right), & \;if\;s_{ij}>0 & LTP\\ w_{ij}\left(t\right)+w_{ij}^{max}f\left(s_{ij}\right), & if\;s_{ij}<0 & LTD \end{array}$$ (5)

Whenever the same pre-synaptic cell fired more than once within the STDP time window, only the first spike was used in STDP calculations [32]. This ‘nearest only’ paradigm is in contrast with the alternative ‘all pairings’ paradigm, whereby all pre-synaptic spikes for each cell take part in STDP [33, 34]. It has been demonstrated that either paradigm results in the same equilibrium state when used with mixed learning [32]. The ‘nearest only’ paradigm was chosen for the simulations described in this paper because it is less computationally expensive.

### Cochlear nucleus (CN)

The model CN was implemented as three parallel cell populations: 1000 CH neurons, 1000 PL neurons and 100 ON neurons. The CH cells removed space jitter while ON cells removed time jitter. Reducing time and space jitter is achieved through the following mechanisms: 1) information from a number of AN fibers with similar characteristic frequencies (CFs) is integrated within the tonotopically tuned CH neurons in order to remove space jitter; and 2) AN spike trains from neurons with a wide range of CFs drive activity within the ON cells which phase locks to the fundamental frequency of the auditory stimulus. This periodic input from the ON cells combined with the inputs from CH and PL subpopulations of the CN then help stabilise the firing within the IC by producing spike rasters with reduced jitter in both space and time (see [10] for details). The modelling choices for the three subpopulations of CN are described in more detail below.

**Fig 3:**
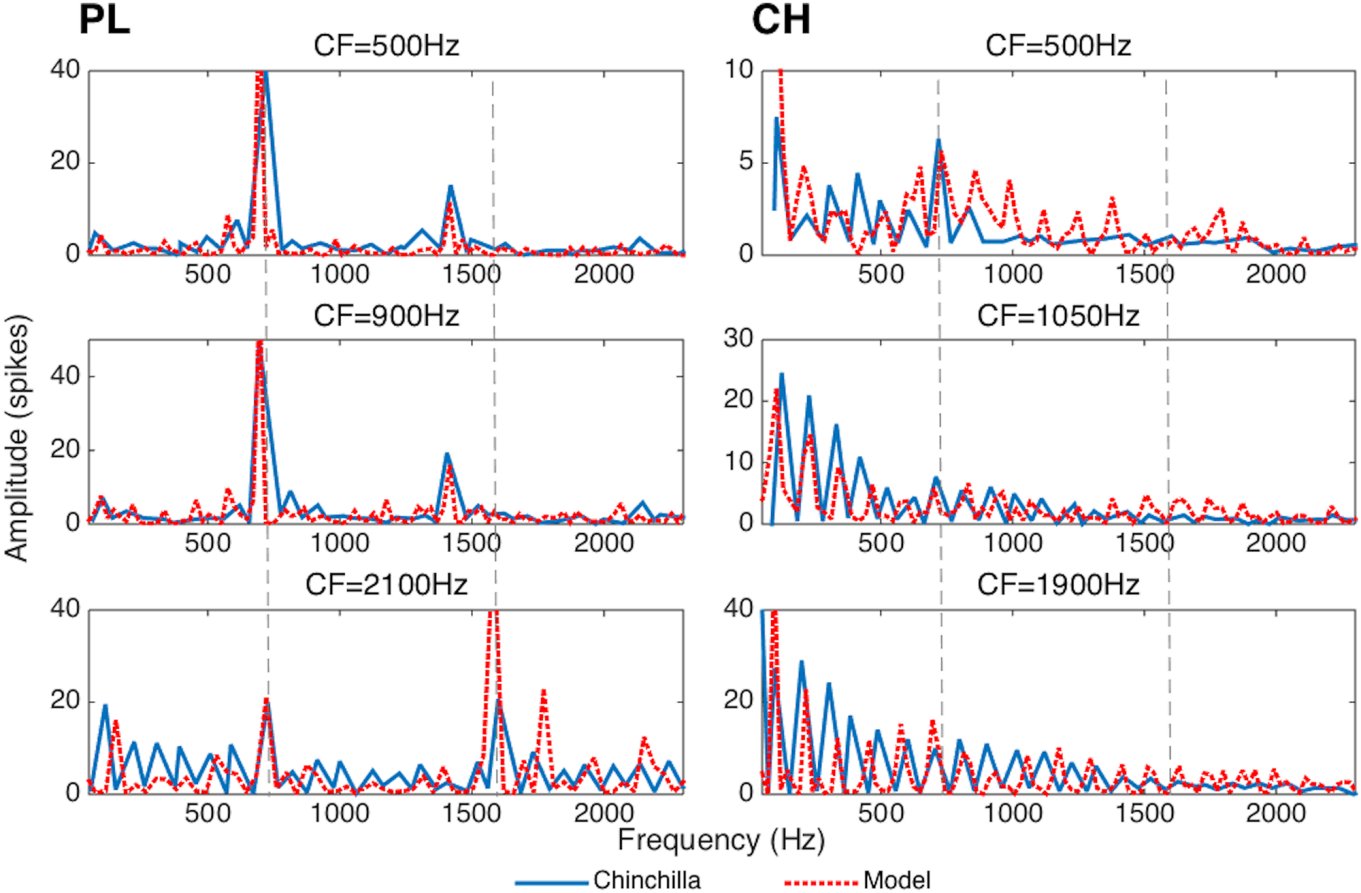
Spectra (computed as Fast Fourier Transforms of period histograms) of primary-like (PL) (left column) and chopper (CH) (right column) cochlear nucleus neuron responses to a synthetic vowel /a/ generated using the Klatt synthesiser [36]. The ordinate represents the level of phase-locking to the stimulus at frequencies shown along the abscissa. Dotted lines show the positions of the vowel formant frequencies *F*_1_ and *F*_2_. Data from chinchilla CN fibers reproduced from [37] is shown in solid blue. Data collected from the corresponding model CN fibers is shown in dashed red. Similarity between the real and model fibers” response properties suggests that the model’s performance is comparable to the neurophysiological data.

In the brain CH cells receive a small number of afferent connections from AN neurons with similar CFs [35]. The incoming signals are integrated to produce regular spike trains. In the full AN-CN-IC-CX model, a CH subpopulation was simulated by units with Gaussian topological input connectivity, where each CH cell received afferents from a small tonotopic region of the AN (*σ* = 26 AN fibers). Their discharge properties correspond closely to those reported experimentally for biological CH neurons (Fig 3, right column).

PL neurons make up ≈ 47% of the ventral CN in the brain [35], suggesting an important role in auditory processing. Although their contribution to the processing of AN discharge patterns is perhaps less clear, informal tests in our model indicate that their inclusion leads to significantly better model performance [10]. PL cells essentially transcribe AN firing [35] and were modelled using strong one-to-one afferent connections ($w_{ij}^{BL}$) from the AN. The discharge properties of the model PL neurons also correspond closely to those reported experimentally (Fig 3, left column).

ON cells are relatively rare, constituting around 10% of the ventral CN [35]. They require strong synchronized input from many AN fibers with a wide range of CFs in order to fire [24], which results in broad frequency tuning and enables them to phase-lock to the fundamental frequency (*F*_0_) of voiced speech sounds [38]. In the full AN-CN-IC-CX model, an ON cell population was simulated using sparse (34%) connectivity from across the AN. The interplay between converging ON and CH cell inputs to the IC can reduce jitter in the neural representation of vocalisation sounds. Since ON cells synchronise to the voice *F*_0_, they can introduce regularly spaced strong afferent input to the IC. Even if these afferent currents are sub-threshold, they nevertheless prime the postsynaptic IC cells to discharge at times corresponding to the cycles of stimulus *F*_0_. If IC cells also receive input from CH cells, then ON afferents will help synchronise CH inputs within the IC by increasing the likelihood of the IC cells firing at the beginning of each *F*_0_ cycle. This is similar to the neural coding ideas first described by [7].

### Inferior colliculus (IC) and auditory cortex (CX)

Each model IC cell received one-to-one afferent connectivity from PL, narrow tonotopic connectivity from CH (*σ* = 2 neurons) and full connectivity from the ON cells. These subcortical connections ($w_{ij}^{BL}$) were not plastic. The IC→A1 and A1→Belt (and the equivalent AN→A1 and A1→Belt in the reduced AN-CX model) connectivity was full, feedforward, with STDP learning [8] (parameters *α*_*p*_, *α*_*d*_, *τ*_*p*_ and *τ*_*d*_ in our models, see Table 1) and a uniform distribution of conduction delays (Δ_*ij*_ ∈ [0, 50] ms) [39]. The initial afferent connection strengths were randomly initialised using values drawn from a uniform distribution $w_{ij}^{BL}\in \left[30,35\right]$ nA.

### Polychronization index

As outlined in the introduction, we envisaged that our model of the auditory brain would exhibit unsupervised learning of speaker independent representations of naturally spoken words “one” and “two” by forming a hierarchy of stable spatio-temporal patterns of firing (PGs). An exhaustive search for PGs through the network was prohibitive, especially given that the number of neurons participating in each PG was unknown a priori, and could be large and variable [40]. We therefore developed a numerical score, the “polychronization index” (PI) to quantify the prevalence of reproducible patterns of firing across the population of neurons in one layer.

The PI was calculated for each cell *j* within a particular stage of the model. For each spike produced by cell *j* we searched for spikes fired by other cells within the same stage of the model within a fixed time interval Δ*t*_*j*_. PGs which are informative of a stimulus class should be reproducible across the different presentations of different exemplars *e*_*k*_(*s*) of the stimulus class, where *k* ∈ {1,…,376} is the number of exemplars (4 repetitions of 94 pronunciations) of each of the *s* = 2 stimulus classes (words “one” and “two”). For each presentation of stimulus exemplar *e*_*k*_(*s*) we randomly selected one spike by cell *j* and noted its time *t*_*j*_ post stimulus onset. We then constructed a 1000 neuron by 101 ms matrix $M_{e_{k}\left(s\right)}^{j}$ representing the firing of the other neurons within the same stage of the model that occured within Δ*t* = *t*_*j*_ ± 50 ms at 1 ms resolution. The ±50 ms time window reflects the maximum conduction delay (Δ_*ij*_) in our model, which puts an upper limit on the temporal integration window of neuron *j*. If neuron *j* is part of a PG selective of a particular stimulus class *s*, then we would expect to see similar firing pattern matrices $M_{e_{k}\left(s\right)}^{j}$ for different *e*_*k*_(*s*). Consequently, elements of $M_{e_{k}\left(s\right)}^{j}$ which are non-zero across the different stimulus exemplars more frequently than would be expected by chance (where chance levels can be estimated from the average firing rate *f*) are diagnostic of the PG firing pattern. The larger the proportion of stimulus exemplars for which these elements are non-zero, the more established and reproducible the PG firing pattern is across the responses to the different exemplars from the given stimulus category.

We therefore computed $M_{s}^{j}=\langle M_{e_{k}\left(s\right)}^{j}\rangle$, where 〈 ⋅ 〉 signifies the mean over all the exemplars *e*_*k*_(*s*) of stimulus class *s*, and then identified the ten elements of $M_{s}^{j}$ with the largest mean spike counts ${\left(m_{s}^{j}\right)}_{n}$, where *n* ∈ {1,…,10} (the element corresponding to the randomly sampled spike by cell *j* occurring at time *t* was ignored). These were used to compute $a_{s}^{j}=\langle {\left(m_{s}^{j}\right)}_{n}\rangle /f$, where *f* is the average firing rate within the layer and 〈 ⋅ 〉 signifies the mean over the ten largest elements indexed by *n*.

Thus, $a_{s}^{j}$ quantifies the evidence that cell *j* participates in polychronous firing in its responses to stimulus class *s*. To calculate an overall polychronization index which is not stimulus specific we simply compute $PI^{j}=max_{s}\left(a_{s}^{j}\right)$. The larger the *PI*^*j*^, the stronger the evidence that cell *j* takes part in a PG.

### Information analysis

Apart from quantifying whether PGs arise throughout the hierarchy of our model of the auditory brain, it is imporant to measure how informative these PGs are about the two stimulus classes, words “one” and “two”. One common way to quantify such learning success is to estimate the mutual information between stimulus class and neural response *I*(*S*; *R*). It is calculated as $I\left(S;R\right)=\sum_{s\in S,r\in R}p\left(s,r\right)log_{2}\frac{p(s,r)}{p(s)p(r)}$, where *S* is the set of all stimuli and *R* is the set of all possible PG responses, *p*(*S*, *R*) is the joint probability distribution of stimuli and responses, and *p*(*s*) = ∑_*r*∈*R*_ *p*(*s*, *r*) and *p*(*r*) = ∑_*s*∈*S*_ *p*(*s*, *r*) are the marginal distributions [41]. Stimulus-response confusion matrices were constructed using decoder multi-layer perceptron (MLP) networks (see below), and used to calculate *I*(*S*;*R*) (see Table 3 for details).

**Table 3:** Joint probability table used to calculate multiple cell information *I*(*S*;*R*). #*PredictStim*1 (#*PredictStim*2) stands for the number of true positives for the word “one” (“two”) produced by the MLP. #*Stim*1 (#*Stim*2) stands for the total number of presentations of the different exemplars of the word “one” (“two”). *N* stands for the total number of stimulus classes (*N* = 2 for the two naturally spoken digit discrimination task).

		Actual	
		**Stimulus 1**	**Stimulus 2**
Predicted	**Stimulus 1**	$\frac{\#PredictStim1}{\#Stim1*N}$	$\frac{\#PredictStim1}{\#Stim2*N}$
	**Stimulus 2**	$\frac{\#PredictStim2}{\#Stim1*N}$	$\frac{\#PredictStim2}{\#Stim2*N}$

Our information analysis approach uses observed frequencies as estimators for underlying probabilities *p*(*s*), *p*(*r*) and *p*(*s*, *r*). This introduces a positive bias to our information estimates $Bias\approx \frac{\#bins}{2Nlog_{2}2}$, where #*bins* is the number of potentially non-zero cells in the joint probability table, and *N* is the number of recording trials [41]. Given the large *N* in our tests of model performance (*N* = 752), the bias was negligible (*Bias* ≈ 0.004 bits) and was ignored.

The upper limit of mutual information to be achieved by PGs in our models *I*(*S*; *R*) is given $H\left(s\right)=\sum_{s}p\left(s\right)log_{2}\frac{1}{p(s)}$, which, given that we had two equiprobable stimulus classes, here equals 1 bit.

Decoder MLPs were used to evaluate the ability of PGs within the full AN-CN-IC-CX and the reduced AN-CX models of the auditory brain to represent stimulus identity using either rate or temporal encoding schemes. The nature of the input feature vectors *x*_*i*_ for the temporal and rate encoding schemes is described below.

**Temporal code**: Temporal encoding assumes that information about stimulus class is provided by spatio-temporal firing patterns (PGs). In order to be informative, the same PG must be present more frequently in response to the exemplars of one stimulus class than the other. The vectors *x*_*i*_ used as input to the decoder MLPs were designed to capture PGs within a particular stage of the auditory brain model. As discussed previously, it is unfeasible to explicitly identify PGs in our models. We do not know which neurons within a stage of the auditory brain model participate in a PG and which ones do not. We therefore introduce an approximate computationally feasible protocol to get an indication of the presence of PGs within a stage of the model, and then to use these approximations to calculate how informative the approximated PGs in this stage are. We appreciate that our meaure is not perfect, but we accept it, because it approximately lowerbounds the informativeness of PGs in the models, and it is informative enough to differentiate between the different models.

Our protocol takes the following form. First, in order to restrict the computational load, we randomly sample *J* = 100 cells from within the chosen auditory model stage. We make an assumption that at least some cells *j* within this sample will take part in a PG. We then train a separate MLP_*j*_ to try and classify the two stimulus classes, words “one” and “two”, using the approximated PGs that each cell *j* is part of. If our assumption was correct and cell *j* indeed participated in a PG, then MLP_*j*_ will achieve high classification accuracy, otherwise the classification accuracy will be low.

We quantified reproducible PG spatio-temporal patterns for each cell *j* using methods analogous to those used to calculate the polychronization index (PI) (see Sec. Polychronization Index). For each cell *j* we first computed a 100 neuron by 101 ms matrix $M_{e_{k}\left(s\right)}^{j}$. This was computed in the same way as for the PI score (see Section Polychronization Index), where 100 neurons are the randomly sampled subset of 100 neurons within the auditory model stage. For each of the 752 input stimuli *e*_*k*_(*s*) we then concatenated together a 100 element vector of row sums and a 101 element vector of column sums of matrix $M_{e_{k}\left(s\right)}^{j}$ to form the 201 element column vectors *x*_*i*_ for training the MLP_*j*_. Summing and concatenating reduces the dimensionality of the feature vectors from 100 * 101 = 10,100 (the dimensionality of matrix $M_{e_{k}\left(s\right)}^{j}$) to 201, while still capturing the key patterns of firing across the sample population of 100 cells in response to a single presentation of stimulus *e*_*k*_(*s*) and the amount of spatio-temporal structure in the network activity that would be compatible with the presence of PGs. Each of the decoders MLP_*j*_ was then trained to classify response vectors *x*_*i*_ as either the word “one” or word “two”.

The confusion matrix used in the information analysis calculations described above was constructed based on the majority classification votes among the 100 trained MLP_*j*_ in response to each stimulus presentation.

The proposed MLP decoding scheme is supposed to be a way to quantitatively approximate the informativeness of the PGs that emerge within the different layers of our model through unsupervised STDP learning. We do not suggest that a decoder like this is implemented in the brain. Instead we believe that over a number of cortical stages the polychronous groups will learn to span an ever larger proportion of the low dimensional data manifolds as shown in Fig 2C, and that this will be reflected in the increasing MI scores computed through using the proposed MLP method.

**Rate code**: A random subset of *J* = 201 cells within an auditory brain model stage was chosen to match the dimensionality of the input vectors *x*_*i*_ for training the MLPs for the temporal code informativeness estimation. The average firing rate of the 201 subsampled neurons was recorded in response to each of the 752 stimulus presentations to form the input to the rate code MLP.

**Using multi-layer perceptron (MLP) decoders to evaluate network performance**: We compared the amount of information about the two stimulus classes “one” and “two” when using either rate or temporal encoding schemes in the two models, the full AN-CN-IC-CX and the reduced AN-CX, using decoder MLPs. The MLPs were trained to classify 752 input vectors *x*_*i*_ ∈ *R*^201^ as either word “one” or “two”. Our MLP decoders had a small, single hidden layer of 20 units (10% of the input layer size) to limit their capacity and therefore to make them more sensitive to the informativeness of different auditory brain models and encoding schemes under investigation. The MLPs had hidden layer neurons with hyperbolic tangent transfer functions. They were trained until convergence using scaled conjugate gradient descent [42] and a cross-entropy loss function. Once the MLPs were trained, their classification outputs were used to fill the confusion matrices used in the information analysis calculations described above. The process was repeated 20 times, each time with a different random subsample of *J* cells, to obtain a distribution of stimulus information estimates for each cell population.

## Results

In this paper we propose that speaker-independent word representations are encoded by unique discriminative spatio-temporal patterns of firing (PGs) in the auditory cortex. We test this hypothesis using a biologically inspired spiking neural network model of the auditory brain. Since we cannot explicitly detect the information bearing PGs due to computational restrictions, we present instead multiple pieces of evidence for the emergence of informative PGs within the plastic cortical stages of our model. These pieces of evidence include the particular change in the distribution of connection weights ($w_{ij}^{BL}$) after training that is characteristic of PG-based learning, the increased polychrony of firing in the final cortical stages of the model (measured by the polychronisation index we devised in Section Polychronization Index), and the performance of MLP decoders trained to be sensitive to the existence of stimulus class selective PGs. The observed differences according to these measures between the full AN-CN-IC-CX and the reduced AN-CX models, and between rate and temporal encoding schemes, provide evidence in support of our hypothesis that more information is carried using temporal PGs rather than rate codes, and that the emergence of such informative PGs is only possible if stable input firing patterns are provided to the plastic cortical stages of our models.

**Fig 4:**
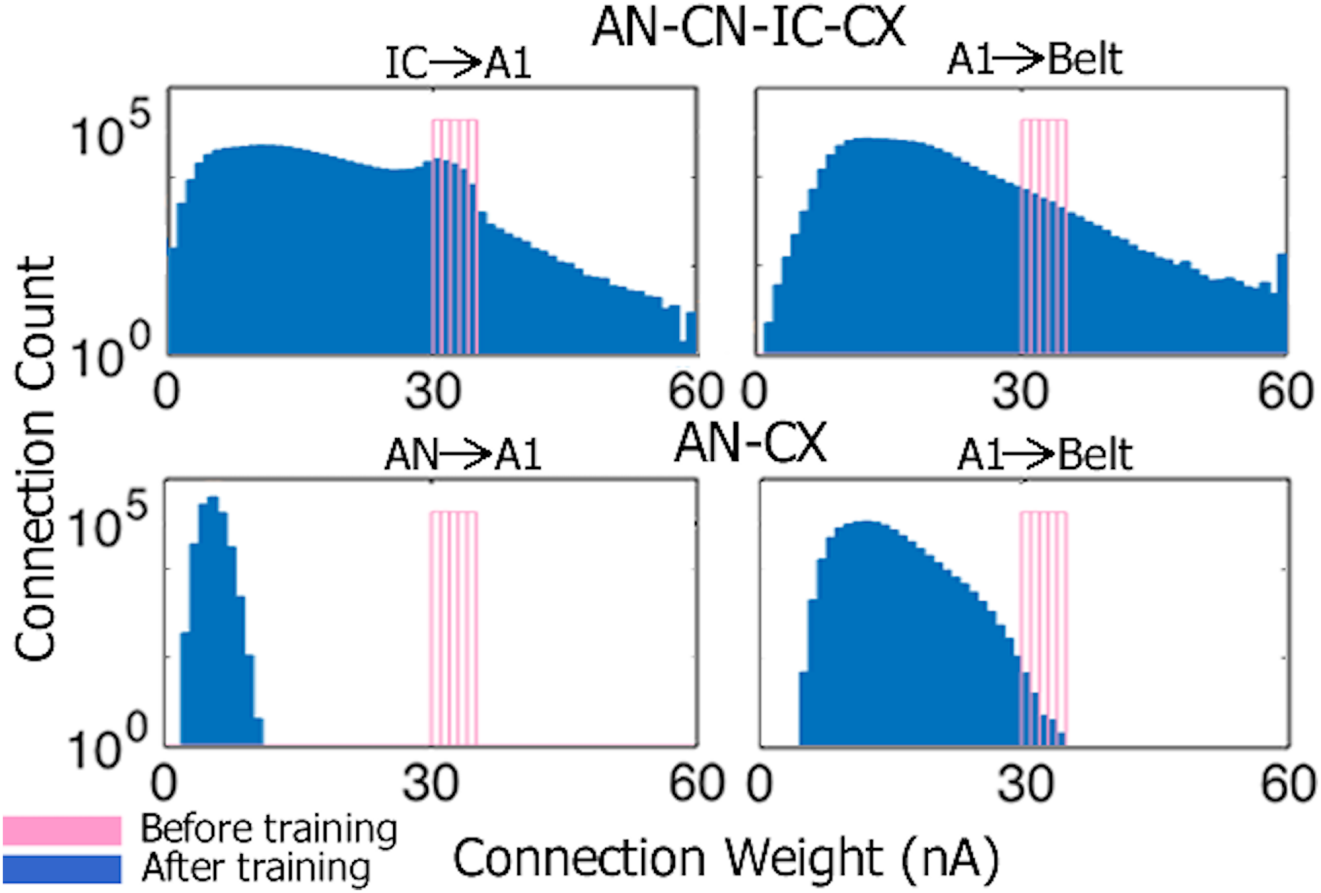
Distributions of synaptic connection weights ($w_{ij}^{BL}$) before (pink) and after (blue) training for the full AN-CN-IC-CX model (top row) and reduced AN-CX model (bottom row). The ordinate is log scaled.

**Changes in synaptic weights resulting from unsupervised learning**: To examine the synaptic weight changes that occurred in the cortical stages of the full AN-CN-IC-CX model during training we plotted the distributions of IC→A1 and A1→Belt connections ($w_{ij}^{BL}$) before and after training (Fig 4, top row). While the majority of weights grew weaker, some connections were strengthened. Such a pattern of change is characteristic of learning PGs. Since the STDP configuration in our model is reminiscent of that described in [5], the majority of connections ($w_{ij}^{BL}$) weakened due the non-informative random firing in the input. Some connections strengthen due to the presence of repeating stable patterns of firing (PGs) among the pre-synaptic neurons.

Compare this pattern of connectivity change to the equivalent stages of the reduced AN-CX model. Almost all AN→A1 and A1→Belt synaptic weights were weakened during training (Fig 4, bottom row). In the reduced model, stochastic jitter in the AN presumably scrambled the structure of input patterns sufficiently to prevent regularities (PGs) in the inputs to be discovered and learned.

These data suggest that PG learning relies on stable patterns of firing that serve as input to the plastic cortical stages A1 and Belt of the models. AN stochasticity hinders such learning, but de-noising of AN firing within the CN and IC makes PG-based learning possible again in the cortical stages of the full AN-CN-IC-CX model.

**Fig 5:**
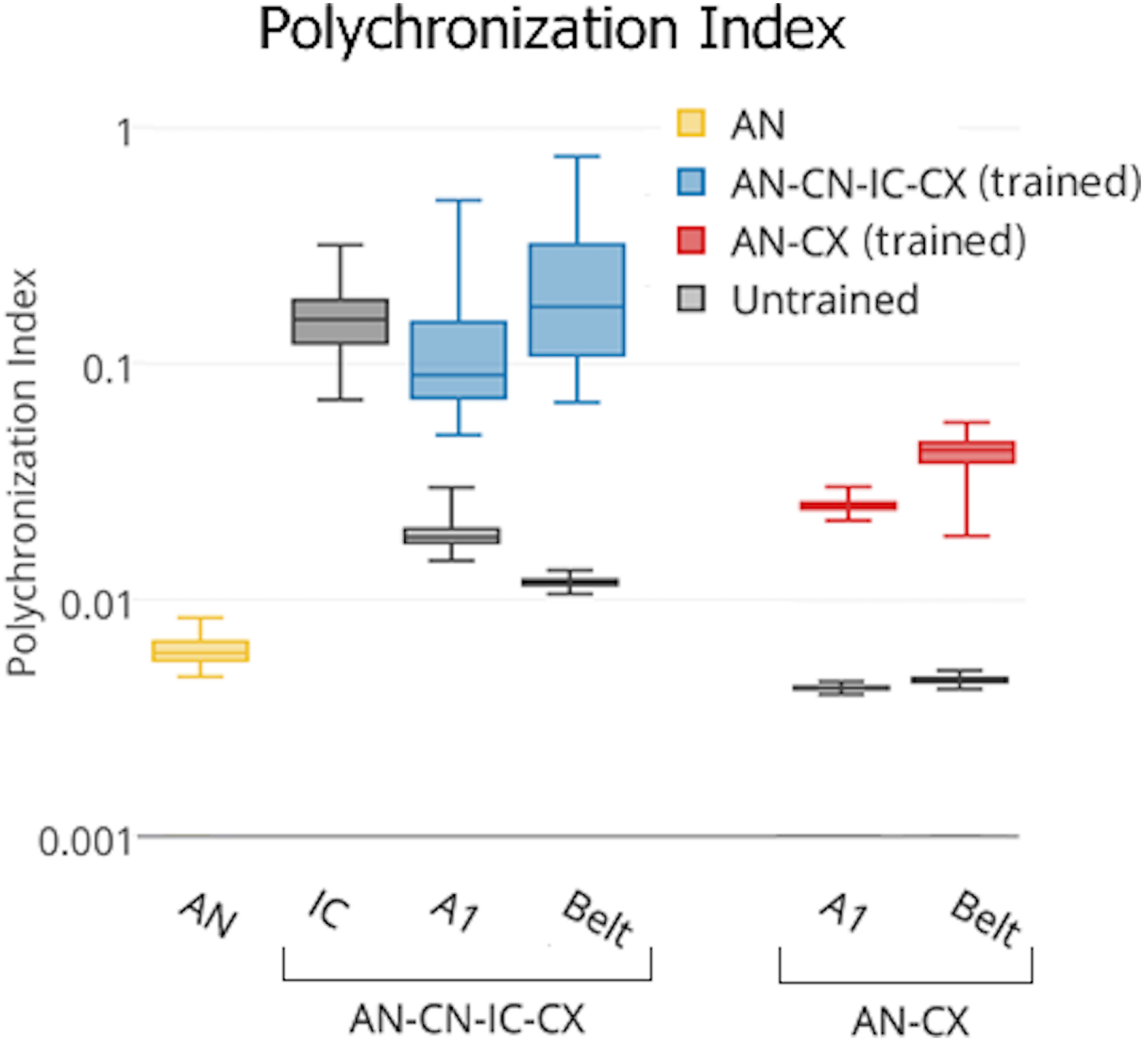
Box-and-whisker plot showing the distribution of polychronization indices over all the cells within the relevant layers of the full AN-CN-IC-CX and reduced AN-CX models. The ordinate is log scaled.

**Denoising of AN firing patterns and the emergence of polychronous groups (PGs)**: Fig 5 shows that subcortical preprocessing in the CN and IC led to more stable spatio-temporal discharge patterns, as evidenced by higher PI scores in the IC compared to AN. These more reproducible firing patterns also carried through to A1 and Belt. As a reminder, PI index only measures how often similar spatio-temporal patterns repeat in response to different auditory stimuli. High PI index means that there are particular combinations of neurons within a layer that have strongly correlated stimulus driven spiking activity. The PI index does not say anything about how informative these stable spatio-temporal firing patters are about the stimulus classes. This is why the PI score is high and stable across the IC, A1 and Belt areas of the trained AN-CN-IC-CX model, despite the fact that the IC is not plastic. All this says is that the CN pre-processing stabilises the noisy AN firing patterns (see the low PI score for the AN) within the IC, which then allows the plastic CX to learn. The AN-CX model, which lacked subcortical preprocessing layers CN and IC, did not achieve the same stability of firing patterns seen in IC and CX of the full model even after training.

**Fig 6:**
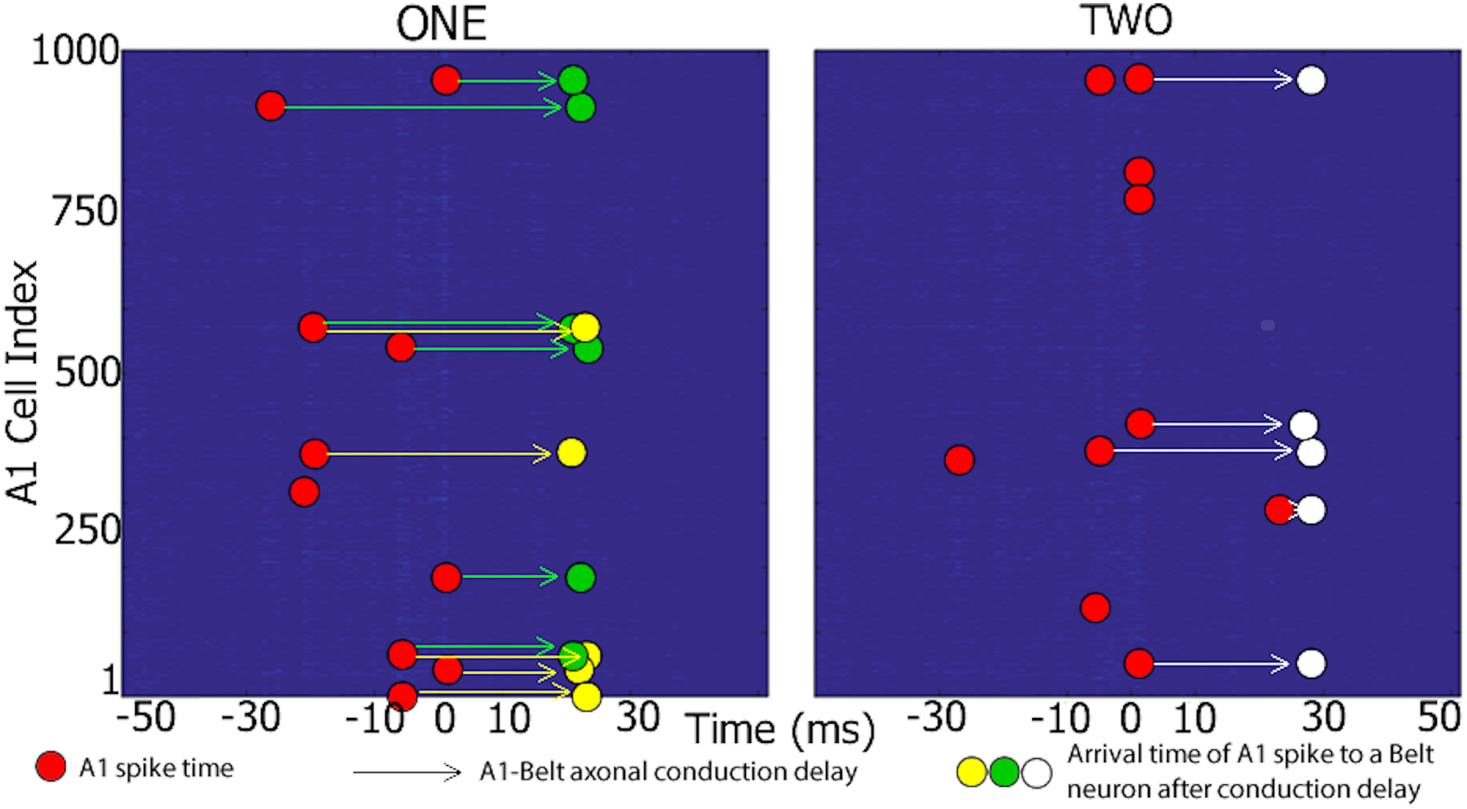
Evidence for PGs responding selectively to word “one” or “two” in the A1 layer of the trained AN-CN-IC-CX model. Each plot shows an example of a stable spatio-temporal spike pattern in A1 (red circles) in response to different pronunciations of the words “one” (left) and “two” (right). These spikes take part in at least one polychronous group that is selective for the particular word. In other words, these patterns are more likely to appear when an example of their preferred word is pronounced compared to an example of a non-preferred word. When projected through the A1→Belt connections ($w_{ij}^{BL}$) with different conduction delays (Δ_*ij*_) (arrows), these patterns produce near-synchronous input from several A1 neurons onto a subset of Belt neurons (green, yellow or white circles corresponding to three separate Belt neurons with different distributions of axonal conduction delays Δ_*ij*_). The green and yellow circles show such inputs for two Belt neurons which in this manner respond selectively for a number of different pronunciations of the word “one”, the white circles show inputs for a neuron that responded selectively to exemplars of the word “two”. Abscissa represents the time window Δ*t* = *t*_*j*_ ± 50 ms around the origin. The origin is centered around all the times *t* when a chosen A1 neuron *j* fires (see Section Polychronization Index for details). Ordinate represents the 1000 neurons that make up A1 in the AN-CN-IC-CX model. Red circles show the ten elements of the firing pattern matrix $M_{s}^{j}$ with the largest mean spike counts ${\left(m_{s}^{j}\right)}_{n}$ (see Section Polychronization Index for details).

**Polychronous groups**: Fig 6 shows a partial visualisation of three PGs that emerged in the A1 layer of the trained AN-CN-IC-CX model. These PGs responded preferentially either to different pronunciations of the word “one” or “two”. The red dots show the ten elements of the mean firing pattern matrix $M_{s}^{j}$ with the largest mean spike counts ${\left(m_{s}^{j}\right)}_{n}$ (see Section Polychronization Index). They can be thought of as partial PGs observed in response to the respective stimulus classes. When projected through the A1→Belt connections ($w_{ij}^{BL}$) and the corresponding axonal delays (Δ_*ij*_), these patterns produce near-synchronous input from four or more A1 neurons in a small subset of Belt neurons, and these inputs are consequently strengthened during training. The green and yellow dots show such inputs for two Belt neurons which in this manner became selective for the word “one”, the white dots show equivalent data for a Belt neuron that became selective for “two”.

**Fig 7:**
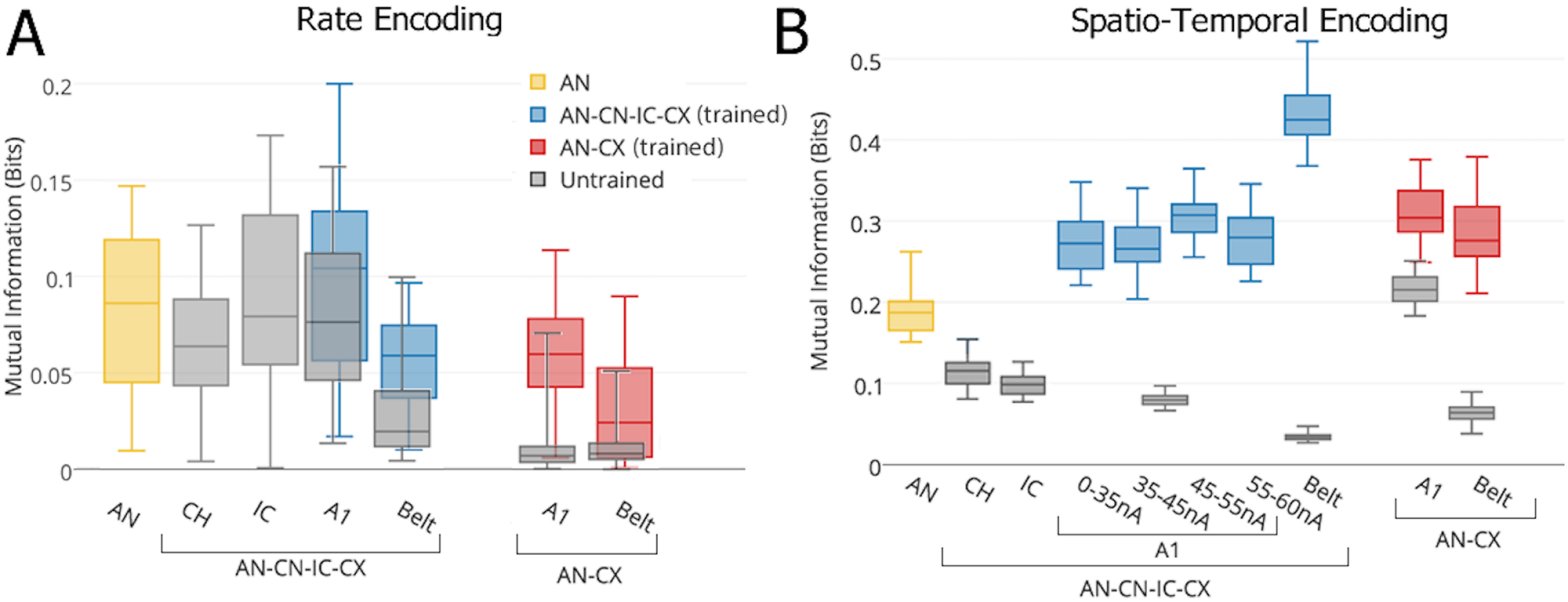
Box-and-whisker plots showing the distribution of information over twenty different subsamples of cells within various layers of the full AN-CN-IC-CX and reduced AN-CX models based on rate (A) and temporal (B) encoding schemes. In **B**, the A1 neurons are subdivided according to the strength of their connections ($w_{ij}^{BL}$) to the Belt layer. PL and ON layers are not shown. The ON layer conveys 0 bits of information, and PL is equivalent to AN.

**Stimulus category encoding across models and layers**: Fig 7 shows estimates of the speech stimulus identity information encoded in various layers of the full AN-CN-IC-CX and the reduced AN-CX models. Note the different y-scale ranges in panels A and B. Temporal encoding provides substantially more stimulus category information than rate encoding at every stage of the model. After training, the responses of the Belt layer of the full model carried as much as 0.52 bits per response. In comparison, in the untrained model, the stimulus category information never exceeded 0.05 bits.

A control simulation was run to test the ability of an untrained AN-CN-IC-CX model with the same distribution of IC-A1 and A1-Belt afferent connection weights ($w_{ij}^{BL}$) as the trained model to discriminate between the two stimulus classes. This was done by randomly shuffling the corresponding connection weights ($w_{ij}^{BL}$) of the trained AN-CN-IC-CX model before presenting it with the two naturally spoken word stimuli. It was found that such a model performed at the same level as the untrained model with afferent connection weights initialised from the $w_{ij}^{BL}\in \left[30,35\right]$ nA uniform distribution (0.05 (shuffled) vs 0.08 (random) bits in the A1, and 0.04 (shuffled) vs 0.03 (random) bits in Belt).

Fig 7B also shows that responses in the plastic A1 and Belt layers of the full AN-CN-IC-CX model contain significantly more stimulus category information after unsupervised learning than the input AN layer responses. This indicates that the biologically inspired spiking AN-CN-IC-CX model has learnt to develop a more efficient, less redundant and more informative representation of the naturally spoken word stimuli during training, in line with [43]. This is achieved using only physiologically realistic, local STDP learning. After learning, the Belt area of the full AN-CN-IC-CX model encoded more than twice as much stimulus category information as the AN, but it failed to reach the maximum of 1 bit of information required for perfect word identification. The information encoding performance of the AN-CN-IC-CX model should be possible to improve by the addition of recurrent plastic within-layer cortical connections or additional plastic cortical layers. We leave this, however, for future work.

When the trained model was tested on additional data of the same words “one” and “two” being spoken by twenty novel speakers (ten male and ten female speakers pronouncing each word twice), the network reached a similar level of performance as described above (0.27 (new data) vs 0.28 (old data) bits in the A1, and 0.48 (new data) vs 0.43 (old data) bits in Belt).

It is interesting to note that those A1 cells which acquired A1→Belt connections in the $w_{ij}^{BL}\in \left[45,55\right]$ nA range are more informative than A1 cells with maximally strengthened connections. This effect is in line with the work by [5]. In order to maximally strengthen the A1→Belt connections ($w_{ij}^{BL}$), PGs in the A1 have to appear very freuquently. This is more likely to happen if the PGs are present in response to both stimulus classes “one” and “two”. Such PGs, however, are not informative of the stimulus class identity. This effect also explains why A1 and Belt stages of the full AN-CN-IC-CX model have higher levels of spatio-temporal stimulus category information than the IC stage of the model despite no increase in the PI from IC upwards. This is because PI score only measures the presence of PGs in a particular stage of the model without measuring their informativeness. The data suggests that while the degree of stability of spatio-temporal firing in the IC, A1 and Belt stages of the full AN-CN-IC-CX model is similar, the stimulus class selectivity and hence the informativness of PGs grows throughout this feedforward hierarchy.

### Overview of PG-based learning

The patterns of MI and PI scores acheived by the different stages of the AN-CN-IC-CX model shown in Figs 5 and 7 match what might be expected from the learning mechanisms described in Section “Learning mechanisms”. The non-plastic subcortical pre-processing upto the IC stage of the AN-CN-IC-CX model denoises the AN input and hence increases the PI score. Since high PI scores only correspond to the presence of stable spatio-temporal patterns of firing (PGs) within a model stage, and do not measure how informative these PGs are about the autitory stimuli, one should not necessarily expect an increase in MI with higher PI scores. And indeedd, we find that the MI score in the IC is lower than that in the AN. This happens because the CN and the IC are not able to increase the informativeness of PGs due to the lack of plasticity within these sub-cortical stages. Instead the denoising capabilities of the different subpopulations of the CN and their convergence within the IC result in the creation of a library of PGs in the IC that are often active in response to both words “one” and “two” and are therefore not very informative. The stimulus specificity and hence the informativeness of such PGs is then improved through learning in the plastic CX stages of the AN-CN-IC-CX model according to the principles described in Section “Learning mechanisms”. This is evidenced by the rapid increase in MI within the CX stages of the AN-CN-IC-CX model. The PI score stays stable within these stages—the stability of PGs is preserved and only their informativeness is increased through learning.

On the other hand, the AN-CX model is not able to achieve the same increase in MI throughout the plastic CX as the AN-CN-IC-CX model due to the lack of PGs in any of its stages as demonstrated by their lower PI scores. CX plasticity in the AN-CX model is able to improve MI scores slightly between the AN and the A1, however this increase is not as large as between the IC and A1 stages of the AN-CN-IC-CX model. Furthermore, MI increase stagnates in the AN-CX model compared to the further increase between the A1 and Belt stages of the AN-CN-IC-CX model.

## Discussion

In this paper we argued that a hierarchy of speaker-independent informative PGs is learnt within the different stages of the plastic cortical layers of the full AN-CN-IC-CX model. The learning in the model, however, is reliant on the input of stable firing patterns to the plastic cortical stages A1 and Belt. Such stable firing patterns are obscured by stochasticity in the raw AN firing rasters [10]. Consequently the cortical layers are essentially unable to learn speaker independent representations of naturally spoken words using unprocessed AN input (reduced AN-CX model). Subcortical preprocessing in the CN and IC stabilises and de-noises the AN firing patterns, thus allowing the cortical ensembles of the full AN-CN-IC-CX model to form category specific response patterns.

Our work builds on the work described by [44, 45], who demonstrated how a simple spiking neural network can robustly differentiate between specific spatio-temporal patterns of inputs. Unlike our work, however, the network described in [44, 45] was not capable of learning in an unsupervised manner through biologically plausible mechanisms, such as STDP learning. Instead the connection weights of their model were manually adjusted for differentiating between different input patterns. The inputs to the model were also less realistic than in our work. Another major difference between our approach and that of [44, 45] is that they rely on the presence of recurrent connections within their model, while the PGs described in our model can develop in both feed-forward (as described in this paper) and recurrent architectures (left for future work, but see [6]).

While we use a physiologically grounded AN model [17] to produce the input spiking activity, [44, 45] used simplified manual pre-processing of the auditory data which lacked the same level of realism and stochasticity as is present in the auditory brain. The biological realism and the stochasticity of the inputs to our model also set our results apart from work by [46], who achieved similar informativeness for differentiating between words “one” and “two” as our model (around 0.6 bits) using a recurrent network of winner-take-all microcircuits with STDP learning. The input to the model by [46], however, were much simpler than those used in our work. While the model by [46] had to differentiate between only three utterances of the words “one” and “two” pronounced by two speakers, our model had to differentiate between to four repetitions of the same words pronounced by 94 speakers.

Another model relevant to the work described in this paper was proposed by [16]. The authors modelled three stages of the sub-cortical auditory brain: the auditory nerve (AN), the chopper units of the ventral cochlear nucleus (CN), the inferior colliculus (IC). These were followed by the higher Level 4 neurons for which the corresponding brain area was not specified. It was shown that the model could accurately replicate many psychophysical results of pitch perception, thus demonstrating the potential computational mechanisms underlying pitch perception in the brain. The architecture of the model suggested by [16] is somewhat similar to the architecture we suggest for our model. Furthermore, the emphasis on the neurophysiological realism of the model in order to gain insights into the computational principles underlying auditory brain function is also shared with our approach. Our model, however, relaxes the strong connectivity assumptions imposed on the model by [16] and shown to be paramount for their model’s operation. In our model the local connectivity for the AN→CN and CN→IC areas is drawn from a Gaussian distribution rather than hard-coded as in [16]. Furthermore, we extend the model by [16] by adding onset and primary-like CN cells, as well as higher order A1 neurons with STDP-based learning. The addition of STDP-based learning in particular makes our model more powerful, whereby it is able to adapt to the underlying structure of the auditory stimuli through long-term STDP-based learning, while the model by [16] lacks any learning capabilities.

In summary, the biological realism of the inputs to our model and the presence of unsupervised biologically plausible STDP learning sets our results apart from the similar work by [16, 44–46] described above.

We took inspiration from the known neurophysiology of the auditory brain in order to construct the spiking neural network models used in this paper. As with any model, a number of simplifying assumptions had to be made with regards to certain aspects that we believed were not crucial for testing our hypothesis. These simplifications included the lack of superior olivary complex or thalamus in our full AN-CN-IC-CX model, the nature of implementation of within-layer inhibition in both the AN-CX and AN-CN-IC-CX models, and lack of top-down or recurrent connectivity in either model. Furthermore, the particular values we chose to parametrise the different Izhikevich neuron types [21] were somewhat arbitrary, since other values could also result in similar functional performance of the neurons and hence in similar qualitative performance of the models. All of the chosen parametrisations, however, were biologically realistic as described in [21]. While all of these simplifications do affect the learning of auditory object categories to some extent, we believe that their particular implementation in our models does not undermine out qualitative findings and conclusions.

The full AN-CN-IC-CX model of the auditory brain described in this paper possesses a unique combination of components necessary to simulate the emergent neurodynamics of auditory categorisation learning in the brain, such as biologically accurate spiking dynamics of individual neurons, axonal conduction delays, STDP learning, neuroanatomically inspired architecture and exposure to realistic speech input. With its biological realism, the full AN-CN-IC-CX model described in this paper can be used to make testable predictions about the auditory object encoding in the auditory brain. For example, the reduction of jitter in spiking responses as one ascends from AN through IC to auditory cortex should be observable in physiological experiments. Furthermore, large channel count recordings may make it possible to discover PG firing patterns which encode categorical stimulus information in the activity of ensembles of real cortical neurons.

## Supporting information

S1 Text (PDF)

S1 Fig Average auditory nerve (AN) and inferior colliculus (IC) firing rasters in response to naturally spoken digits “one” and “two” pronounced by 94 speakers 4 times each. The abscissa represents time (ms), while the ordinate represents cell index within the corresponding layer. The cells are tonotopically organised to match the layout of S2 Fig. Each firing raster is accompanied by a histogram indicating the total number of spikes for each neuron in response to all the exemplars of the corresponding stimulus class as shown in the raster.
(TIFF)

S2 Fig Spectrograms (right) and auditory nerve (AN) firing rasters (left) in response to naturally spoken digit ‘one’ pronounced by speaker 1 and naturally spoken digit ‘two’ pronounced by speaker 3. The abscissa of both types of graphs represents time (ms), while the ordinate represents the log frequency (Hz) in the spectrograms and the AN cell index for the firing rasters. The characteristic frequencies (CFs) of the AN fibers roughly correspond to the horizontally aligned frequencies in the spectrograms. It can be seen that the AN firing rasters roughly match their corresponding spectrograms. It can also be seen that the speaker fundamental frequencies are different for the two speakers, which is represented by the larger gaps between the vertical lines of concurrent AN cell firing in the ‘one’ firing raster compared to the ‘two’ firing raster. Furthermore, the vowel onset time happens later in response to digit ‘two’ compared to digit ‘one’, as evidenced by the later onset of firing within the AN fibers with lower CFs.
(TIFF)

S3 Fig Typical auditory nerve (AN) and inferior colliculus (IC) firing rasters in response to naturally spoken digits “one” and “two” pronounced by one of the 94 speakers. The abscissa represents time (ms), while the ordinate represents cell index within the corresponding layer. The cells are tonotopically organised to match the layout of S2 Fig. Each firing raster is accompanied by a histogram indicating the total number of spikes for each neuron in response to all the exemplars of the corresponding stimulus class as shown in the raster.
(TIFF)

S4 Fig Average A1 and Belt firing rasters in response to noise stimuli generated by randomly permuting auditory nerve spikes per time step in response to digits “one” and “two” pronounced by 94 speakers 4 times each. The abscissa represents time (ms), while the ordinate represents cell index within the corresponding layer. The cells are tonotopically organised to match the layout of S2 Fig. Each firing raster is accompanied by a histogram indicating the total number of spikes for each neuron in response to all the exemplars of the corresponding stimulus class as shown in the raster.
(TIFF)

## References

[1] Huetz C, Gourevitch B, Edeline J. Neural codes in the thalamocortical auditory system: From artificial stimuli to communication sounds. Hearing Res. 2011;271:147–158. 10.1016/j.heares.2010.01.010 20116422

[2] Bizley J, Walker K, King A, Schnupp J. Neural ensemble codes for stimulus periodicity in auditory cortex. J Neurosci. 2010;30(14):5078–5091. 10.1523/JNEUROSCI.5475-09.2010 20371828 PMC2864913

[3] Frisina R. Subcortical neural coding mechanisms for auditory temporal processing. Hearing Res. 2001;158:1–27. 10.1016/S0378-5955(01)00296-9 11506933

[4] Evans B, Stringer S. Transformation-invariant visual representations in self-organizing spiking neural networks. Front Comput Neurosci. 2012;6(46):1–19. 22848199 10.3389/fncom.2012.00046 PMC3404434

[5] Masquelier T, Guyonneau R, Thorpe S. Spike Timing Dependent Plasticity Finds the Start of Repeating Patterns in Continuous Spike Trains. PLoS ONE. 2008;3(1). 10.1371/journal.pone.0001377 18167538 PMC2147052

[6] Izhikevich E. Polychronization: Computation with Spikes. Neural Comput. 2006;18 10.1162/089976606775093882 16378515

[7] Hopfield J. Pattern recognition computation using action potential timing for stimulus representation. Nature. 1995;. 10.1038/376033a0 7596429

[8] Bi GQ, Poo MM. Synaptic modifications in cultured hippocampal neurons: dependence on spike timing, synaptic strength, and postsynaptic cell type. J Neurosci. 1998;18 9852584 10.1523/JNEUROSCI.18-24-10464.1998 PMC6793365

[9] Leonard R, Doddington G. TIDIGITS speech corpus. Texas Instruments Inc 1993;.

[10] Higgins, I, Stringer, S, Schnupp, J. Auditory Nerve Stochasticity Impedes Auditory Category Learning: a Computational Account of the Role of Cochlear Nucleus and Inferior Colliculus in Stabilising Auditory Nerve Firing. biorxiv. 2016; 10.1101/059428. 29652586

[11] Stringer S, Perry G, Rolls E, Proske J. Learning invariant object recognition in the visual system with continuous transformations. Bioll Cybern. 2006;94:128–142. 10.1007/s00422-005-0030-z 16369795

[12] Liao, Q, Leibo, JZ, Poggio, T. Learning invariant representations and applications to face verification. NIPS. 2013;.

[13] Tromans JM, Higgins IV, Stringer SM. Learning View Invariant Recognition with Partially Occluded Objects. Frontiers in Computational Neuroscience. 2012;6(48). 10.3389/fncom.2012.00048 22848200 PMC3404435

[14] Rhode WS, Roth GL, Recio-Spinoso A. Response properties of cochlear nucleus neurons in monkeys. Hearing Research. 2010;259:1–15. 10.1016/j.heares.2009.06.004 19531377 PMC2815100

[15] Evans EF. Auditory Processing of Complex Sounds: An Overview Reviewed. Philosophical Transactions: Biological Sciences. 1992;336(1278):295–306. 1354368 10.1098/rstb.1992.0062

[16] Meddis R, O’Mard LP. Virtual pitch in a computational physiological model. J Acoust Soc Am. 2006;120(6):3861–3869. 10.1121/1.2372595 17225413

[17] Zilany M, Bruce I, Nelson P, Carney L. A phenomenological model of the synapse between the inner hair cell and auditory nerve: long-term adaptation with power-law dynamics. Acoust J Soc Am. 2009;126(5). 10.1121/1.3238250 PMC2787068 19894822

[18] Eggermont J. Between sound and perception: reviewing the search for a neural code. Hearing Res. 2001;157:1–42. 10.1016/S0378-5955(01)00259-3 11470183

[19] Wever E, Bray C. The nature of acoustical response: the relation between sound frequency and frequency of impulses in the auditory nerve. J Exper Psychol. 1930;13.

[20] Izhikevich E. Simple Model of Spiking Neurons. IEEE Trans Neural Netw. 2003;14(6). 10.1109/TNN.2003.820440 18244602

[21] Izhikevich EM. Which Model to Use for Cortical Spiking Neurons? IEEE Transactions on Neural Networks. 2004;15(5):1063–1070. 10.1109/TNN.2004.832719 15484883

[22] Goodman DF, Brette R. Brian: a simulator for spiking neural networks in Python. Front Neuroinform. 2008;2(5). 10.3389/neuro.11.005.2008 19115011 PMC2605403

[23] Hawkins J, Ahmad S. Why Neurons have thousands of synapses, a theory of sequence memory in neocortex. Frontiers in Neural Circuits. 2016;. 10.3389/fncir.2016.00023 PMC4811948 27065813

[24] Oertel D, Bal R, Gardner S, Smith P, Joris P. Detection of synchrony in the activity of auditory nerve fibers by octopus cells of the mammalian cochlear nucleus. PNAS. 2000;97(22). 10.1073/pnas.97.22.11773 11050208 PMC34348

[25] Ulanovsky N, Las L, Farkas D, Nelken I. Multiple time scales of adaptation in auditory cortex neurons. J Neurosci. 2004;24:10440–10453. 10.1523/JNEUROSCI.1905-04.2004 15548659 PMC6730303

[26] Tikidji-Hamburyan R, Martinez J, White J, Canavier C. Resonant interneurons can increase robustness of gamma oscillations. Journal of Neuroscience. 2015;35(47):15682–15695. 10.1523/JNEUROSCI.2601-15.2015 26609160 PMC4659828

[27] Wang XJ, Buzsaki G. Gamma oscillation by synaptic inhibition in a hippocampal interneuronal network model. Journal of Neuroscience. 1996;16:6402–13. 8815919 10.1523/JNEUROSCI.16-20-06402.1996 PMC6578902

[28] Deneve S, Machens CK. Efficient codes and balanced networks. Nature Neuroscience. 2016;. 10.1038/nn.4243 26906504

[29] Perrinet L, Delorme A, Samuelides M, Thorpe SJ. Networks of integrate-and-fire neu- ron using rank order coding A: how to implement spike time dependent hebbian plasticity. Neurocomputing. 2001;38-40:817–822. 10.1016/S0925-2312(01)00460-X

[30] Debanne D, Gahwiler BH, Thompson SM. Cooperative interactions in the induction of long-term potentiation and depression of synaptic excitation between hippocampal CA3-CA1 cell pairs in vitro. Proc Natl Acad Sci USA. 1996;93:11225–11230. 10.1073/pnas.93.20.11225 8855337 PMC38312

[31] Debanne D, Gahwiler BH, Thompson SM. Heterogeneity of synaptic plasticity at unitary CA1-CA3 and CA3-CA3 connections in rat hippocampal slice cultures. J Neurosci. 1999;19:10664–10671. 10594050 10.1523/JNEUROSCI.19-24-10664.1999 PMC6784957

[32] van Rossum MCW, Bi GQ, Turrigiano GG. Stable Hebbian Learning from Spike Timing-Dependent Plasticity. Journal of Neuroscience. 2000;20(23):8812–8821. 11102489 10.1523/JNEUROSCI.20-23-08812.2000 PMC6773092

[33] Kempter R, Gerstner W, van Hemmen JL. Hebbian learning and spiking neurons. Phys Rev E. 1999;59:4498–4514. 10.1103/PhysRevE.59.4498

[34] Song S, Miller KD, Abbott LF. Competitive Hebbian learning through spike-timing-dependent synaptic plasticity. Nat Neurosci. 2000;3:919–926. 10.1038/78829 10966623

[35] Winter I, Palmer A. Responses of single units in the anteroventral cochlear nucleus of the guinea pig. Hearing Res. 1990;44:161–178. 10.1016/0378-5955(90)90078-4 2329092

[36] Klatt DH. Speech perception: a model of acoustic-phonetic analysis and lexical access In: Cole RA, editor. Perception and production of fluent speech. Hillsdale, NJ: Lawrence Erlbaum Associates; 1980.

[37] Recio A, Rhode W. Representation of vowel stimuli in the ventral cochlear nucleus of the chinchilla. Hearing Res. 2000;146:167–184. 10.1016/S0378-5955(00)00111-8 10913893

[38] Winter I, Palmer A, Wiegrebe L, Patterson R. Temporal coding of the pitch of complex sounds by presumed multipolar cells in the ventral cochlear nucleus. Speech Commun. 2003;41 10.1016/S0167-6393(02)00098-5

[39] Miller R. Axonal conduction times and human cerebral laterality A psychobiological theory. Harwood Academic; 1996.

[40] Paugam-Moisy H, Martinez R, Bengio S. Delay Learning and Polychronization for Reservoir Computing. Neurocomputing. 2008;71(7-9):1143–1158. 10.1016/j.neucom.2007.12.027

[41] Nelken I, Chechik G. Information theory in auditory research. Hearing Res. 2007;229 10.1016/j.heares.2007.01.012 17300891

[42] Moller M. A scaled conjugate gradient algorithm for fast supervised learning. Neural Netw. 1993;6(4). 10.1016/S0893-6080(05)80056-5

[43] Barlow HB. Possible Principles Underlying the Transformations of Sensory Messages. Sensory Communication. 1961;1.

[44] Hopfield JJ, Brody CD. What is a moment? “Cortical” sensory integration over a brief interval. PNAS. 2000;97:13919–13924. 10.1073/pnas.250483697 11095747 PMC17676

[45] Hopfield JJ, Brody CD. What is a moment? Transient synchrony as a collective mechanism for spatiotemporal integration. PNAS. 2001;98:1282–1287. 11158631 10.1073/pnas.031567098 PMC14746

[46] Klampfl S, Maass W. Emergence of Dynamic Memory Traces in Cortical Microcircuit Models through STDP. The Journal of Neuroscience. 2013;. 10.1523/JNEUROSCI.5044-12.2013 23843522 PMC6618695

