# Supplementary material for: Unsupervised learning of temporal features for word categorization in a spiking neural network model of the auditory brain
Source: PLoS One. 2017 Aug 10;12(8):e0180174. doi: 10.1371/journal.pone.0180174 (PMC5552261; doi:10.1371/journal.pone.0180174)
Supplement: S1 Text — (PDF) [file pone.0180174.s001.pdf]

Unsupervised Learning of Temporal Features for Word Categorization in a Spiking Neural Network Model of the Auditory Brain

Irina Higgins[1*], Simon Stringer[1], Jan Schnupp[2]

1 Department of Experimental Psychology, University of Oxford, Oxford, England

2 Department of Physiology, Anatomy and Genetics (DPAG), University of Oxford, Oxford, England

* irina.higgins@gmail.com

Supplementary Materials

To gain a better intuition of what is happening in the full AN-CN-IC-CX model we plot the average firing rate within the AN and IC stages of the trained model in response to words ”one” and ”two” spoken by 94 different speakers four times each (Fig 1). It can be seen that the firing rate drops within the IC compared to the AN, which corresponds to the denoising effect of the subpopulations of the CN described in the paper. Furthermore, while overall the patterns of firing in response to the two stimuli are hard to differentiate based on these aggregated firing rasters due to between- and within-speaker variability, our results demonstrate that informative PGs can be learnt from the IC responses. The average firing rasters look much blurrier for the AN compared to the IC, which provides further support for our hypothesis that the subpopulations of the CN and their convergence in the IC helps denoise AN input and encourage the development of PGs. There is still certain level of noise present in the visualised average IC firing raster because it is unlikely that the same PGs will appear at exactly the same time post stimulus onset due to the inherent variability of speech production.

Example spectrograms of digit ’one’ spoken by speaker 1 and of digit ’two’ spoken by speaker 3, as well as their resulting AN firing rasters can be seen in S 2 Fig. It can be

Fig 1. Average auditory nerve (AN) and inferior colliculus (IC) firing rasters in response to naturally spoken digits "one" and "two" pronounced by 94 speakers 4 times each. The abscissa represents time (ms), while the ordinate represents cell index within the corresponding layer. The cells are tonotopically organised to match the layout of S 2 Fig. Each firing raster is accompanied by a histogram indicating the total number of spikes for each neuron in response to all the exemplars of the corresponding stimulus class as shown in the raster.

Fig 2. Spectrograms (right) and auditory nerve (AN) firing rasters (left) in response to naturally spoken digit 'one' pronounced by speaker 1 and naturally spoken digit 'two' pronounced by speaker 3. The abscissa of both types of graphs represents time (ms), while the ordinate represents the log frequency (Hz) in the spectrograms and the AN cell index for the firing rasters. The characteristic frequencies (CFs) of the AN fibers roughly correspond to the horizontally aligned frequencies in the spectrograms. It can be seen that the AN firing rasters roughly match their corresponding spectrograms. It can also be seen that the speaker fundamental frequencies are different for the two speakers, which is represented by the larger gaps between the vertical lines of concurrent AN cell firing in the 'one' firing raster compared to the 'two' firing raster. Furthermore, the vowel onset time happens later in response to digit 'two' compared to digit 'one', as evidenced by the later onset of firing within the AN fibers with lower CFs.

seen that the AN firing rasters roughly reflect their corresponding sound spectrograms, and that the vowel onset time, which results in the first major burst of activity within the AN fibers with lower characteristic frequencies, is different for the two vowels. It can also be seen that the fundamental frequencies of the two digits are different for the two different speakers. This is evidenced by the larger gap between the columns of concurrent spikes in the AN firing raster for digit 'one' compared to that for digit 'two'.

S 3 Fig demonstrates the average firing rasters of words "one" and "two" spoken by a single speaker four times. It can be seen that the IC emphasises particular features present in the AN, while discarding noise. Furthermore, it can be seen that for a particular speaker it may be possible to differentiate between the two words based on rate information (e.g. presence of firing activity in the middle frequency band indicates word "one"), however this information is not stable across different speakers (see S 1 Fig).

Finally, S 4 Fig demonstrates that the model shows different response properties to noise auditory stimuli that match the average rate of firing within the auditory nerve (AN) input, but lack the spectro-temporal structure of naturally spoken digits 'one' and 'two'. The noise stimuli were generated by permuting the identity of AN fiber for each

Fig 3. Typical auditory nerve (AN) and inferior colliculus (IC) firing rasters in response to naturally spoken digits ”one” and ”two” pronounced by one of the 94 speakers. The abscissa represents time (ms), while the ordinate represents cell index within the corresponding layer. The cells are tonotopically organised to match the layout of S 2 Fig. Each firing raster is accompanied by a histogram indicating the total number of spikes for each neuron in response to all the exemplars of the corresponding stimulus class as shown in the raster.

Fig 4. Average A1 and Belt firing rasters in response to noise stimuli generated by randomly permuting auditory nerve spikes per time step in response to digits ”one” and ”two” pronounced by 94 speakers 4 times each. The abscissa represents time (ms), while the ordinate represents cell index within the corresponding layer. The cells are tonotopically organised to match the layout of S 2 Fig. Each firing raster is accompanied by a histogram indicating the total number of spikes for each neuron in response to all the exemplars of the corresponding stimulus class as shown in the raster.

spike within each time bin. It can be seen that the model is not able to differentiate between the two digits in this case, achieving 0.0677 (A1) and 0.0327 (Belt) bits of mutual information compared to 0.28 (A1) and 0.43 (Belt) bits of mutual information in response to the original naturally spoken digits ‘one’ and ‘two’.
